# The efficacy and safety of direct-acting antiviral regimens for end-stage renal disease patients with HCV infection: a systematic review and network meta-analysis

**DOI:** 10.3389/fpubh.2023.1179531

**Published:** 2023-09-29

**Authors:** Ruochan Chen, Yinghui Xiong, Yanyang Zeng, Xiaolei Wang, Yinzong Xiao, Yixiang Zheng

**Affiliations:** ^1^Key Laboratory of Viral Hepatitis of Hunan, Department of Infectious Diseases, Xiangya Hospital, Central South University, Changsha, China; ^2^National Clinical Research Center for Geriatric Disorders, Xiangya Hospital, Central South University, Changsha, China; ^3^Department of Respiration, Hunan Children's Hospital, Changsha, China; ^4^Hunan Provincial Center for Disease Control and Prevention, Hunan Workstation for Emerging Infectious Disease Control and Prevention, Chinese Academy of Medical Sciences, Changsha, China; ^5^Burnet Institute, St Vincent's Hospital, University of Melbourne, Melbourne, VIC, Australia

**Keywords:** hepatitis (C) virus, end-stage renal disease, direct-acting antiviral, Bayesian Markov Chain Monte Carlo, network meta-analysis

## Abstract

**Background:**

Hepatitis C virus (HCV) infection is an independent risk factor associated with adverse outcomes in patients with end-stage renal disease (ESRD). Due to the wide variety of direct-acting antiviral regimens (DAAs) and the factor of renal insufficiency, careless selection of anti-hepatitis C treatment can lead to treatment failure and safety problems. The integrated evidence for optimized therapies for these patients is lacking. This study would conduct comparisons of different DAAs and facilitate clinical decision-making.

**Methods:**

We conducted a systematic literature search in multiple databases (PubMed, Ovid, Embase, Cochrane Library, and Web of Science) up to 7 August 2023. Study data that contained patient characteristics, study design, treatment regimens, intention-to-treat sustained virologic response (SVR), and adverse event (AE) data per regimen were extracted into a structured electronic database and analyzed. The network meta-analysis of the estimation was performed by the Bayesian Markov Chain Monte Carlo methods.

**Results:**

Our search identified 5,278 articles; removing the studies with duplicates and ineligible criteria, a total of 62 studies (comprising 4,554 patients) were included. Overall, the analyses contained more than 2,489 male individuals, at least 202 patients with cirrhosis, and no less than 2,377 patients under hemodialysis. Network meta-analyses of the DAAs found that receiving ombitasvir (OBV)/paritaprevir (PTV)/ritonavir (R) plus dasabuvir (DSV), glecaprevir (G)/pibrentasvir (P), and sofosbuvir (SOF)/ledipasvir (LDV) ranked as the top three efficacy factors for the HCV-infected ESRD patients. Stratified by genotype, the G/P would prioritize genotype 1 and 2 patients with 98.9%−100% SVR, the SOF/DCV regimen had the greatest SVR rates (98.7%; 95% CI, 93.0%−100.0%) in genotype 3, and the OBV/PTV/R regimen was the best choice for genotype 4, with the highest SVR of 98.1% (95% CI, 94.4%−99.9%). In the pan-genotypic DAAs comparison, the G/P regimen showed the best pooled SVR of 99.4% (95% CI, 98.6%−100%). DAA regimens without Ribavirin or SOF showed the lowest rates of AEs (49.9%; 95% CI, 38.4%−61.5%) in HCV-infected ESRD patients.

**Conclusion:**

The G/P could be recommended as the best option for the treatment of pan-genotypic HCV-infected ESRD patients. The OBV/PTV/R plus DSV, SOF/Velpatasvir (VEL), SOF/Ledipasvir (LDV), and SOF/DCV would be reliable alternatives for HCV treatment with comparable efficacy and safety profiles.

**Systematic review registration:**

https://www.crd.york.ac.uk/prospero/#searchadvanced, PROSPERO: CRD42021242359.

## Introduction

Chronic kidney disease (CKD) patients, especially those under maintenance hemodialysis, are at increased risk of hepatitis C virus (HCV) infection (1). The prevalence of CKD in HCV patients is significantly higher than that in the general population (2–4). HCV infection is an independent risk factor for accelerated CKD progression and is associated with adverse outcomes in patients with end-stage renal disease (ESRD), including hepatic-related hospitalizations, mortality, and poor health-related life quality (2, 3).

The advent of direct-acting antiviral regimens (DAAs) has transformed the treatment of HCV in patients with CKD. There is poor evidence comparing and assessing the efficacy and safety of DAAs in ESRD. The guidelines recommend that HCV-infected CKD patients should be assessed for DAA therapy, with the specific regimen determined by HCV genotype, viral load, treatment history, estimated glomerular filtration rate (eGFR), hepatic fibrosis stage, kidney and liver transplant candidacy, and after consideration of drug–drug interactions (4, 5). Although the sustained virologic response (SVR) rate could even reach 90%−100% with few adverse events (AEs) (4), the choice of DAAs for patients with ESRD has not been elucidated. Renal clearance is the major elimination pathway of Sofosbuvir (SOF), so SOF-based regimens have been questioned for use in ESRD patients. Nevertheless, based on several studies on the safety and efficacy of SOF-based regimens in patients with severe CKD, the Drug Administration of most countries has removed the restricted label for renal impairment. A variety of DAAs are permitted for the treatment of HCV infection in patients with impaired renal function. The comparisons of different DAA regimens applied in HCV-infected ESRD patients are not completely understood, which is necessary to steer guideline development and clinical practice. Moreover, pan-genotypic DAAs simplified the treatment algorithm and supported the campaign to eliminate HCV infection all over the world. Whether these drugs still have excellent performance in ESRD remains to be further confirmed.

To guide best practices for DAAs in patients with CKD and chronic hepatitis C, we performed this systematic review and network meta-analysis to quantify the efficacy and safety of different DAAs for the treatment of HCV-infected ESRD patients. To understand which specific interventions work best, their effects should be explored separately and compared against those of other regimens using the two alternative Bayesian models that can accommodate disconnected networks. The study will facilitate informed clinical decision-making and drafting of HCV treatment guidelines.

## Methods

We performed the systematic review and network meta-analysis according to PRISMA guidelines and prospectively registered on PROSPERO (registration ID: CRD42021242359, https://www.crd.york.ac.uk/prospero/#searchadvanced) (6).

### Literature search

Databases including PubMed, Ovid (BIOSIS Previews Embase), Cochrane Library, and Web of Science were systematically searched under the direction of a medical librarian. The final search was completed on 7 August 2023. The bibliographies of relevant studies and reviews were scrutinized for any additional eligible studies not covered by the literature search. The literature search combined the terms and descriptors related to DAA, HCV, and ESRD concerning literature published in English (details of the searching strategy are available in Supplementary File 1). Conference abstracts and comments were not considered.

### Study selection

Citations were merged together in the Microsoft Access Database to facilitate management. Two researchers (Ruochan Chen and Yinghui Xiong) independently screened articles by title and abstract and further identified them with full-text screening. Non-uniform opinions reached a consensus through discussions with the third researcher (Yanyang Zeng). Both clinical trials and cohort studies were considered and eligible for analysis. The included studies met the following criteria: (1) ESRD patients with HCV infection treated with DAA medication, (2) ESRD patients have an exact definition of CKD stage 4 or 5, and (3) definite DAA regimens were executed in the study. We excluded studies in which (1) no result was specified for ESRD patients, (2) no SVR or AEs were reported, (3) no results were specified for all-oral DAA regimens, and (4) same dataset was used in other included studies.

### Outcome measures

The primary outcome of the study was the mean estimated probability of SVR in various studied DAA regimens for HCV-infected ESRD patients. The SVR was defined as a sustained virologic response at 12 weeks after the end of therapy (SVR12) for patients in the treatment group. The relative rank of efficacy would be calculated by network meta-analysis. For secondary outcomes, the AEs reported in the studies, particularly the serious adverse events (SAEs), discontinuation of treatment, or death, were extracted. AEs evaluation included physical examinations, clinical laboratory tests, and symptoms. SAEs were defined as any event leading to hospital admission or resulting in death, or any event considered serious in the opinion of the treating physician.

### Data extraction

Study characteristics (first author, publication year, location, study design, study period), SVR, and AE data per regimen were extracted into a structured electronic database, while two researchers (Ruochan Chen and Yinghui Xiong) completed a cross-check procedure. The Methodological Index for Non-Randomized Studies (MINORS) and the Newcastle-Ottawa Score (NOS) were used to assess the quality of trials and cohort studies, respectively (literature evaluations are available in Supplementary File 2) (7, 8). Disagreements were resolved by consensus and arbitration by a panel of other investigators within the review team (Yanyang Zeng and Yixiang Zheng).

### Statistical analysis

The network meta-analysis in this study would be regarded as a “disconnected network,” while many single-arm studies of DAA were included. On account of the promising efficacy and safety results, the FDA updated their 2017 guidance to industry on the design and analysis of clinical trials of DAAs to recommend the use of single-arm/historical controls as well as a placebo-deferred trial design (9).

The disconnected network analysis was conducted according to the National Institute for Health and Clinical Excellence Guideline (10). With neither direct comparisons nor a common comparator through which to derive indirect comparisons of comparator treatments, the evidence base will be structured as a disconnected network. The Bayesian Markov Chain Monte Carlo (MCMC) method was used to estimate the pooled SVR of each DAA regimen. The random-effects model with binomial likelihood was implemented to predict the distribution of baseline and treatment effects in the analysis. Bayesian models could accommodate disconnected networks; assuming that the variance of the baseline response is fixed at zero, we applied the absolute response as a means to evaluate the efficacy and safety of the regimens. All the studied DAA regimens were respectively combined to estimate the probability of SVR with a 95% equal tail credible interval (95% CI). Relative ranks of the efficacy of different DAA regimens were established in the analysis. Primary calculations were done according to modified intention to treat (mITT) analysis, where only patients who received at least one dose of DAAs and had an assessment of HCV RNA at 12 weeks after completion of treatment were included for SVR analysis. Additional sensitivity analysis was done using intention to treat (ITT). For ITT, all patients who received at least one dose of DAA regimens were analyzed. Subgroup analyses were pre-specified to separate the distinct kinds of HCV genotype, CKD stage, cirrhosis, and hemodialysis. We checked whether the MCMC procedure had reached convergence by visually inspecting the history trace plots and the autocorrelation plots for irregularities. Since the included articles were almost single-arm studies, a single-rate meta-analysis with a random-effects model was used for safety evaluation and subgroup analyses. All the statistical analyses were performed using WinBUGs (version 1.4.3) and R version 4.1.0.

## Results

### Characteristics of included studies

**Figure 1:**
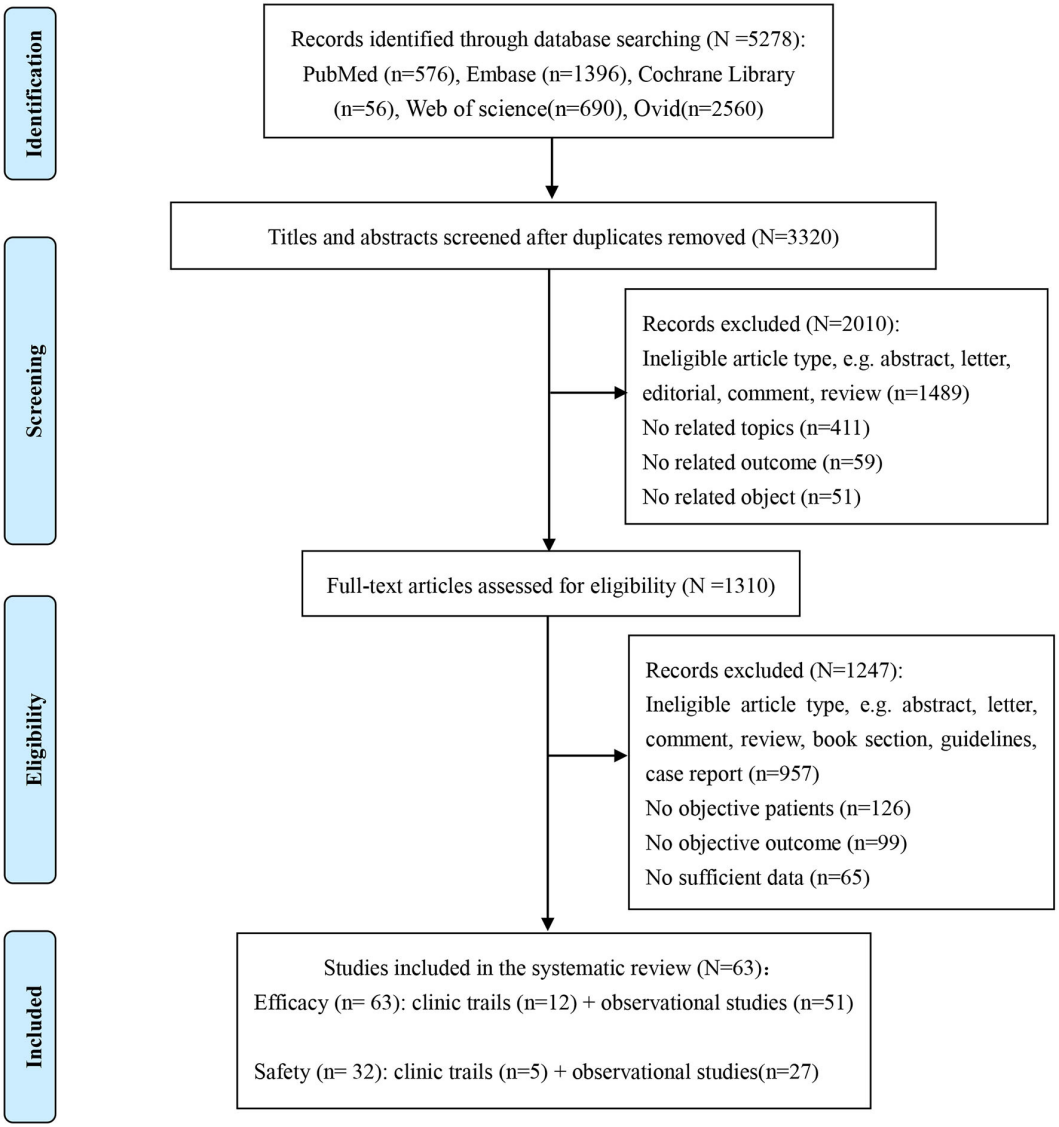
Study selection flowchart summarizing the selection and identification of trials and studies.

Our systematic search yielded 5,278 identified articles; after duplicates and ineligible article types were removed, 62 articles (11–72) (12 clinical trials and 51 observational cohorts) were selected from the 1,310 full-text articles review (Figure 1). One of the included articles from Lawitz et al. (44) reported two cohort studies (RUBY-I, Cohort-2 NCT002207088, and RUBY-II NCT02487199). The included studies were conducted in 27 countries and published between 2015 and 2023. A total of 4,554 HCV-infected ESRD patients who reported the SVR were included in the network meta-analysis for efficacy. Overall, the analyses contained more than 2,485 men, at least 461 patients with cirrhosis, and no < 2,421 patients under hemodialysis. The genotypes of HCV in the study ranged from genotype 1–6, and 1,855 genotype 1 patients, 170 genotype 2 patients, 142 genotype 3 patients, and 150 genotype 4 patients were reported for analysis. Meanwhile, the safety meta-analysis included 32 studies involving 2,176 HCV-infected ESRD patients (11–15, 19–26, 30, 33–37, 39, 45, 47, 48, 51, 57, 59, 60, 62, 64, 66, 68, 69). The safety assessment of all-oral DAAs reported 889 AEs, including 162 SAEs, 38 discontinuations, and 23 deaths.

**Figure 2:**
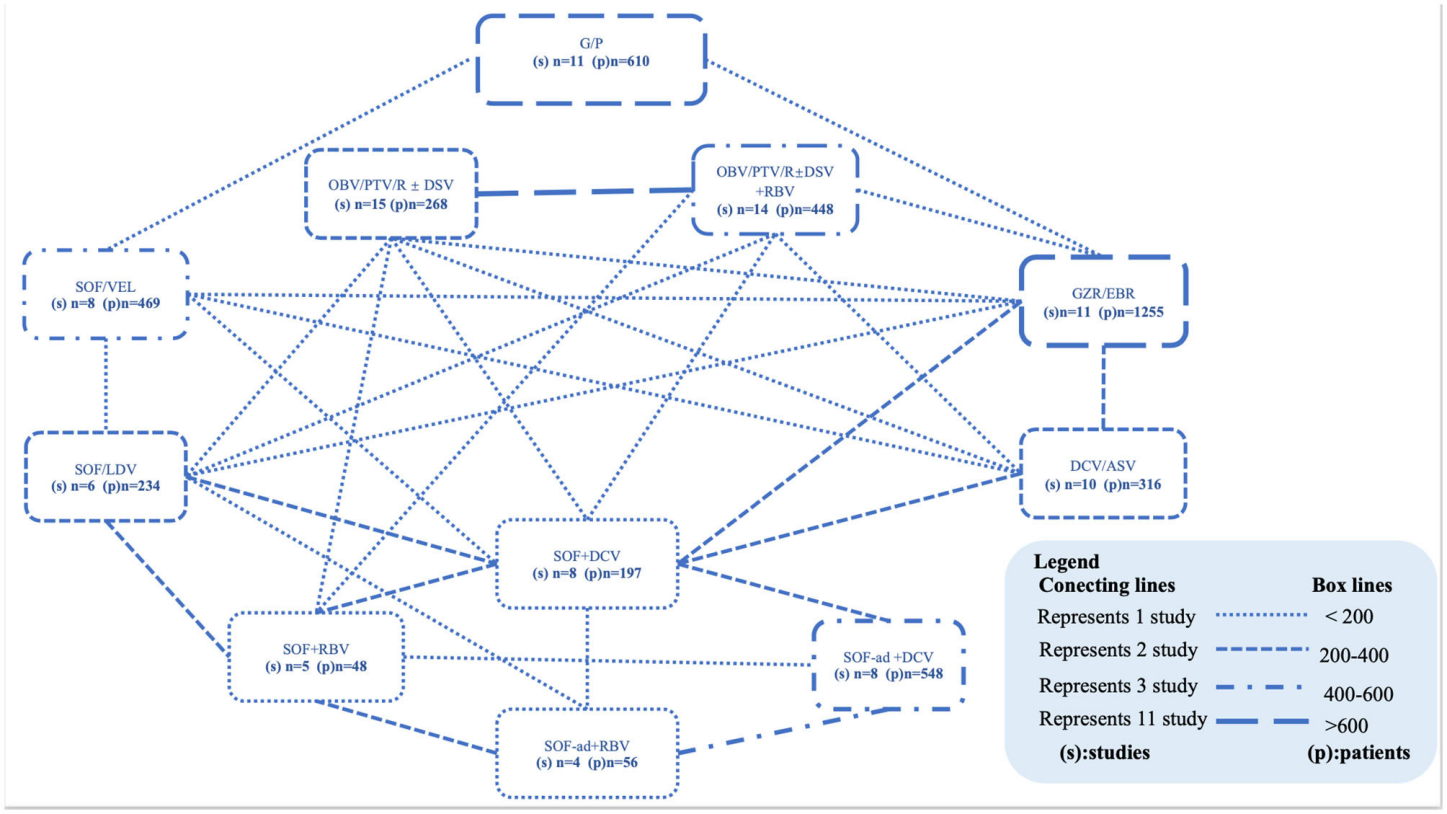
Networks of studies. Evidence network of all DAA-based regimens studied in end-stage renal disease patients with HCV infection. The thickness of the lines represents the number of studies (connecting lines) or the total number of patients studied (box lines). Within the box, the DAA combinations with the number of studies and number of patients are visible. ASV, asunaprevir; DCV, daclatasvir; DSV, dasabuvir; G/P, glecaprevir/pibrentasvir; GZR/EBR, grazoprevir-elbasvir; LDV, ledipasvir; OBV, ombitasvir; PTV/R, paritaprevir/ritonavir; RBV, ribavirin; SOF, sofosbuvir; VEL, velpatasvir.

Excluding the Sofosbuvir/Velpatasvir plus Ribavirin, Sofosbuvir/Ledipasvir plus Ribavirin, and Elbasvir/Grazoprevir plus Ribavirin regimens because only a minority of patients received these regimens in one study. We finally included 11 combinations of DAAs, with or without the addition of Ribavirin (daclatasvir/asunaprevir, DCV/ASV; glecaprevir/pibrentasvir, G/P; grazoprevir-elbasvir, GZR/EBR; sofosbuvir/daclatasvir, SOF/DCV; sofosbuvir-adjusted-dose/daclatasvir, SOF-ad/DCV; sofosbuvi + ribavirin, SOF + RBV; sofosbuvir-adjusted-dose plus ribavirin, SOF-ad + RBV; sofosbuvir/ledipasvir, SOF/LDV; sofosbuvir/velpatasvir, SOF/VEL; ombitasvir/paritaprevir/ritonavir plus or not dasabuvir, OBV/PTV/R ± DSV; OBV/PTV/R ± DSV plus ribavirin, OBV/PTV/R ± DSV + RBV) with treatment durations ranging from 8 to 24 weeks. A network was designed to connect these regimens as shown in Figure 2. Further characteristics of the included studies and patients are provided in Table 1.

**Table 1:** Characteristics of included studies and patients.

**References**	**Year**	**Study duration**	**Region**	**Registered No.**	**Study design**	**Intervention**	**Age (range)**	**SVR mITT**	**SVR ITT**	**TN**	**Male (*N*)**	**Genotype**	**Cirrhosis**	**Hematodialysis**
Roth et al. (11)	2015	2014.03.30–2014.11.28	USA, Argentina, Australia, Canada, Estonia, France, Israel, South Korea, Lithuania, Netherlands, Spain, and Sweden	The C-SURFER study, NCT02092350	Phase 3 randomized study of safety and observational study of efficacy	Grazoprevir/ elbasvir	–	115/116	115/122	122	92	1a/1b	7	92
Kawakami et al. (12)	2016	2014.12–2016.01	Japan	UMIN000015539	Exploratory, prospective, multicenter, pilot study	Daclatasvir plus asunaprevir	68 (47–82)	18/18	18/18	18	14	1b	3	18
Miyazaki and Miyagi (13)	2016	2014.11–2015.08	Japan	–	Observational study	Daclatasvir plus asunaprevir	67.9 (59–74)	10/10	10/10	10	7	1b	–	10
Pockros et al. (14)	2016	2014.09.23–2015.02.18	USA	NCT02207088. RUBY-I, Cohort 1	Single-arm, multicenter study	OBV/PTV/r ± DSV + RBV	60 (49–69)	11/12	11/13	20+	17	1a/1b	–	–
						OBV/PTV/r ± DSV		7/7	7/7					
Suda et al. (15)	2016	2015.01–2015.11	Japan	UMIN000016355	Prospective, observational, multicenter study	Daclatasvir plus asunaprevir	63.0 (50–79)	19/20	20/21	21	16	1a/1b	4	21
Toyoda et al. (16)	2016	2014.12–2015.02	Japan	UMIN 000017023	Multicenter, open-label, clinical trial	Daclatasvir plus asunaprevir	65.5 ± 9.5	28/28	28/28	28	16	1b	11	28
Abad et al. (17)	2017	–	Spain	–	Multicentric observational study	OBV/PTV/r ± DSV + RBV	53.3 ± 7.9	17/17	17/17	35	24	1a/1b/4	7	18
						OBV/PTV/r ± DSV		18/18	18/18					
Agarwal et al. (18)	2017	2015.06–2016.09	India	–	Observational study	Sofosbuvir (dose adjustment) plus RBV	33.8 ± 10.2 (16–53)	37/39	37/39	62	41	1/2/3/4/6	–	62
						Sofosbuvir plus RBV		2/2	2/2					
						Sofosbuvir (dose adjustment) plus daclatasvir		6/6	6/6					
						Sofosbuvir plus daclatasvir		14/15	14/15					
Atsukawa et al. (19)	2017	–	Japan	–	Prospective multicenter study	OBV/PTV/r ± DSV	6,431 (49–85)	30/31	30/31	31	25	1b	10	31
Gane et al. (20)	2017	2015.12.21–2016.03.25	Australia, Belgium, Canada, France, Greece, Italy, New Zealand, the United Kingdom, and the United States	NCT02651194	Multicenter, open-label, phase 3 trial	Glecaprevir/ pibrentasvir	57 (28–83)	102/103	102/104	104	79	1/2/3/4/5/6	20	–
Morisawa et al. (21)	2017	2015.12–2016.03	Japan	–	Observational study	OBV/PTV/r ± DSV	66.8 (53–82)	8/10	8/10	10	5	1b	–	10
Munoz-Gomez et al. (22)	2017	2015.04–2015.10	Spain	–	Retrospective, non-interventional, multicenter study	OB OBV/PTV/r ± DSV + RBV	56.1 ± 9.5	20/21	20/21	46	30	1/4	17	–
						OBV/PTV/r ± DSV		24/25	24/25					
Otsuka et al. (23)	2017	2014.12–2015.12	Japan	UMIN000015882	Multicenter prospective trial	Daclatasvir plus asunaprevir	65 (46–86)	21/23	21/23	23	18	1b	–	23
Sperl et al. (24)	2017	2015.04–2016.04	Czech Republic	—	Observational study	Sofosbuvir (dose adjustment) plus daclatasvir	39 (25–53)	6/6	6/6	6	6	3	–	6
Alric et al. (25)	2018	2015	France	—	Multicenter cohort study	Grazoprevir/ elbasvir	58.6 ± 12.7 (24–90)	87/90	87/93	93	55	1/4	14	67
Butt et al. (26)	2018	–	USA	Electronically Retrieved Cohort of HCV Infected Veterans (ERCHIVES)	National cohort	Sofosbuvir/ ledipasvir	–	78/83	78/107	257	–	1/2/3/4/5/6	–	–
						Sofosbuvir/ ledipasvir plus RBV		25/25	25/30					
						OBV/PTV/r ± DSV		42/42	42/55					
						OB OBV/PTV/r ± DSV + RBV		41/46	41/65					
Fujii et al. (27)	2018	2012–2016	Japan	Japanese Red Cross Liver Study Group	Retrospective cohort study	Daclatasvir plus asunaprevir	65 (58–70)	64/67	64/67	67	44	1a	–	67
Gupta et al. (28)	2018	2015.10–2016.10	India	—	Observational study	Sofosbuvir (dose adjustment) plus RBV	48.4 ± 14.5	1/1	1/1	7	5	1a/1b	2	–
						Sofosbuvir (dose adjustment) plus daclatasvir		5/6	5/6					
Kumada et al. (29)	2018	2016.02.22–2016.06.01	Japan	CERTAIN-1	Phase 3, open-label, multicenter study	Glecaprevir/ pibrentasvir	69 (54–78)	12/12	12/12	12	6	1a/2	2	4
Manoj et al. (30)	2018	2015.09–2016.04	India	NCT02563665	Observational cohort study	Sofosbuvir plus RBV	42 (22–80)	26/26	26/26	71	–	1/3	17	11
						Sofosbuvir/ ledipasvir		26/26	26/26					
						Sofosbuvir plus daclatasvir		19/19	19/19					
Ogawa et al. (31)	2018	—	Japan	The Kyushu University Liver Disease Study (KULDS)	Multicenter, real-world cohort study	Grazoprevir/ elbasvir	–	27/30	27/30	30	–	1a/1b	–	20
Sanai et al. (32)	2018	−2017.02	Saudi Arabia	Systematic Observatory Liver Disease (SOLID) registry	Observational cohort study	OBV/PTV/r plus DSV plus RBV	45.7 ± 12.7	54/54	54/54	67	33	1a/1b/4	14	–
						OBV/PTV/r plus DSV		13/13	13/13					
Sperl et al. (33)	2018	2015.07–2016.08	Czech Republic	—	Retrospective study	OB OBV/PTV/r ± DSV + RBV	53.7 (22–69)	7/7	7/7	23	18	1a/1b	6	19
						OBV/PTV/r ± DSV		16/16	16/16					
Suda et al. (34)	2018	2014.11–2016.03	Japan	UMIN000024227	Nationwide retrospective study	Daclatasvir plus asunaprevir	65 (40–83)	118/123	118/123	123	78	1a/1b	5	123
Taneja et al. (35)	2018	2016.01–2016.08	India	—	Observational cohort study	Sofosbuvir (dose adjustment) plus daclatasvir	42.9 ± 13	65/65	65/65	65	40	1a2	21	54
Atsukawa et al. (36)	2019	2016.11–2017.12	Japan	UMIN000029262	*Post-hoc* analysis of a multicenter study	Grazoprevir/ elbasvir	–	37/37	37/37	37	–	1b	–	20
Atsukawa et al. (37)	2019	2017.11–2018.06	Japan	UMIN000032073	Prospective, multicenter study	Glecaprevir/ pibrentasvir	68 (38–88)	140/141	140/141	141	101	1/2/3	41	100
Aydin et al. (38)	2019	2016.06–2018.05	Turkey	—	Real-life retrospective study	OBV/PTV/r ± DSV	57.8 ± 10.5	18/18	18/18	20	18	1/3/4	–	20
						OB OBV/PTV/r ± DSV + RBV		1/1	1/1					
						Sofosbuvir plus RBV		1/1	1/1					
Borgia et al. (39)	2019	2017.04.19–2018.02.28	Canada, the United Kingdom, Spain, Israel, New Zealand, and Australia	NCT03036852	Phase II, open-label single-arm study	Sofosbuvir/ velpatasvir	60 (33–91)	56/58	56/59	59	35	1/2/3/4/6	17	59
Butt et al. (40)	2019	2017.01–2018.12	Pakistan	—	Real-life retrospective study	Sofosbuvir 400 mg/daclatasvir 60 mg no RBV	36.52 ± 10.90	27/31	27/31	31	11	1/3	–	31
Cheema et al. (41)	2019	2017.08.01–2018.04.30	Pakistan	IRCT201706 14034526N3	Prospective, open-label, parallel, non-randomized interventional trial	Sofosbuvir plus daclatasvir	47.22 ± 14.17	15/18	15/18	36	22	1/3	6	36
						Sofosbuvir (dose adjustment) plus daclatasvir	53.89 ± 14.11	14/18	14/18					
Elmowafy et al. (42)	2019	—	Egypt	—	Prospective, single-center study	OBV/PTV/r ± DSV	40.28 ± 10.9	10/10	10/10	34	23	4	–	34
						OB OBV/PTV/r ± DSV + RBV	43.1 ± 11.2	23/24	23/24					
Goel et al. (43)	2019	2015.12–2017.09	India	—	Observational study	Sofosbuvir (dose adjustment) plus daclatasvir	48 (19–75)	37/41	37/41	41	25	1/3/4	5	–
Lawitz et al. (44)	2019	2015.09.21–2015.12.04	USA	RUBY-I, Cohort 2, NCT002207088	Phase 3b, open-label, multi-center studies	OB OBV/PTV/r ± DSV + RBV	57 (32–76)	35/37	35/37	48	40	1a/1b	15	–
						OBV/PTV/r ± DSV		11/11	11/11					
Lawitz et al. (44)	2019	2016.01.21–2016.04.05	USA	RUBY-II, NCT02487199	Phase 3b, open-label, multi-center studies	OBV/PTV/r ± DSV	57 (31–76)	17/18	17/18	18	12	1a/4	–	–
Lee et al. (45)	2019	2016.02–2017.04	Korea	NCT02580474	Open-label, multicenter, interventional, prospective single-arm study	Daclatasvir plus asunaprevir	59 (39–82)	16/20	16/21	21	13	1b	4	21
Maduell et al. (46)	2019	2014.04–2017.03	Spain	–	Prospective, observational, single-center study	Daclatasvir plus asunaprevir	53.6 ± 8.3	2/2	2/2	19	13	1/2/3/4	8	19
						Grazoprevir/ elbasvir		5/5	5/5					
						OB OBV/PTV/r ± DSV + RBV		8/8	8/8					
						OBV/PTV/r ± DSV		1/1	1/1					
						Sofosbuvir plus daclatasvir		3/3	3/3					
Mekky et al. (57)	2019	2017.01–2018.01	Egypt	NCT03341988	Prospective multicenter cohort study	OB OBV/PTV/r ± DSV + RBV	–	72/75	72/75	75	52	4	8	75
Suda et al. (48)	2019	2017.11–2018.06	Japan	UMIN 000031090	Prospective, observational, multicenter study	Glecaprevir/ pibrentasvir	65 (49–77)	26/27	26/27	27	19	2	13	27
Tatar et al. (49)	2019	2016.08–2017.05	Turkey	–	Observational study	OB OBV/PTV/r ± DSV + RBV	51.4 ± 12.1	20/20	20/20	33	23	1a/1b	–	33
						OBV/PTV/r ± DSV	55.6 ± 13.9	13/13	13/13					
Yaraş et al. (50)	2019	2016.07–2017.10	Turkey	–	Observational study	OBV/PTV/r ± DSV	56.03 ± 11.83	22/22	22/22	25	15	1a/1b	–	25
						OB OBV/PTV/r ± DSV + RBV		3/3	3/3					
Abd-Elsalam et al. (51)	2020	2018.01–2018.09	Egypt	–	Observational, open-label prospective study	OB OBV/PTV/r ± DSV + RBV	62 (28–75)	101/103	101/103	103	54	–	–	–
Choi et al. (52)	2020	2016.02.01–2017.08.31	USA	VA Corporate Data Warehouse	Retrospective cohort study	Grazoprevir/ elbasvir	–	625/644	714/740	740	727	1a/1b	–	563
Debnath et al. (53)	2020	2017.01–2018.07	India	–	Single-center, prospective, open-label observational study	Sofosbuvir/ ledipasvir	39.4 ± 8.3	13/13	13/13	18	14	1/2/3	–	18
						Sofosbuvir plus daclatasvir		5/5	5/5					
Eletreby et al. (54)	2020	2014.02–2018.07	Egypt	–	Real-life multicenter cohort study	Sofosbuvir (dose adjustment) plus RBV	–	4/6	4/6	353	–	–	–	–
						Sofosbuvir (dose adjustment) plus daclatasvir		338/347	338/347					
Gaur et al. (55)	2020	2017.06–2018.06	India	–	Retrospective study	Sofosbuvir/ velpatasvir	39.8 ± 10.8	30/31	30/31	31	7	1/3	–	31
Gohel and Borasadia (56)	2020	2017.06.01–2018.02.28	India	–	Single-center, prospective, open-label study	Sofosbuvir/ ledipasvir	–	39/40	39/40	43	29	1/3	–	–
						Sofosbuvir/ velpatasvir		3/3	3/3					
Lawitz et al. (57)	2020	2013.10.07–2017.10.29	USA and New Zealand	NCT01958281	Phase 2b, open-label, non-randomized, multicenter study	Sofosbuvir (dose adjustment) plus RBV	64 (52–70)	4/10	4/10	38	26	1/3	6	–
						Sofosbuvir plus RBV	59 (45–75)	6/10	6/10					
						Sofosbuvir/ ledipasvir	59 (32–66)	18/18	18/18					
Lawitz et al. (70)	2020	2017.03.28–2018.06.05	Canada, Germany, Greece, Italy, Poland, Puerto Rico, South Korea, Spain, Sweden, and the United States	NCT03069365	Phase 3b, open-label, non-randomized, multicenter study.	Glecaprevir/ pibrentasvir	–	74/75	74/77	77	–	1/2/3/4/6	–	77
Li et al. (58)	2020	2018.06–2020.02	China	–	Retrospective observational study	Sofosbuvir plus daclatasvir	50.54 ± 11.27	3/3	3/3	24	15	1/2	–	24
						Daclatasvir plus asunaprevir		3/3	3/3					
						Grazoprevir/ elbasvir		15/15	15/16					
						Sofosbuvir/ velpatasvir		2/2	2/2					
Liu et al. (59)	2020	2018.06–2019.04	China	–	One-arm, open-label, multicenter study	Grazoprevir/ elbasvir	64 (32–85)	38/38	38/40	40	23	1b	–	40
Liu et al. (60)	2020	2018.08–2019.03	China	–	Retrospective study	Glecaprevir/ pibrentasvir	64 (32–87)	107/107	107/108	108	63	1/2/3/6	35	–
Morishita et al. (61)	2020	2017.11–2018.06	Japan	–	Retrospective multicenter study	Glecaprevir/ pibrentasvir	—	24/24	24/24	24	16	1b/2	14	24
Mostafi et al. (71)	2020	2018.10–2019.09	Bangladesh	–	Prospective study	Sofosbuvir (dose adjustment) plus Daclatasvir	43.70 ± 12.01	26/26	26/26	70	30	–	–	70
						Sofosbuvir/ velpatasvir		44/44	44/44					
Poustchi et al. (62)	2020	2017.04–2018.09	Iran	NCT03063879	Multicenter cohort study	Sofosbuvir plus daclatasvir	50.3 ± 13.5	94/94	94/103	103	76	1/2/3/4	39	–
Seo et al. (63)	2020	2017.02–2018.02	Korea	–	Retrospective study	Sofosbuvir plus RBV	65 (27–82)	9/9	9/9	9	6	2	2	9
Stein et al. (64)	2020	2016.07.29–2019.06.30	German	DRKS00009717	Prospective national real-world registry	Glecaprevir/ pibrentasvir	–	29/31	29/33	93	66	1/2/3/4	–	70
						Grazoprevir/ elbasvir		50/56	50/56					
Yap et al. (65)	2020	2017–2018	China	–	Prospective study	Glecaprevir/ pibrentasvir	–	18/19	18/21	21	–	2/3/6	–	–
Yen et al. (66)	2020	2018.08–2019.12	China	–	Retrospective study	Glecaprevir/ pibrentasvir	67.6 ± 12.1	42/42	42/44	44	26	1/2/3/4/6	14	–
Yu et al. (72)	2020	2019.05–2020.04	China	NCT03803410 and NCT03891550	Real-world observatory study	Sofosbuvir/ velpatasvir	65.9 ± 9.6	95/102	95/106	146	71	1/2/6/	37	146
						Grazoprevir/ elbasvir		8/9	8/9					
						Sofosbuvir/ ledipasvir		2/2	2/2					
						Glecaprevir/ pibrentasvir		27/29	27/29					
Cheng et al. (67)	2021	2017.08–2018.12	China	–	Real-world multicenter observatory study	Grazoprevir/ elbasvir	–	107/107	107/107	107	–	1	–	–
Liu et al. (68)	2021	2019.07–2020.03	China	–	Real-world multicenter observatory study	Sofosbuvir/ velpatasvir	64 (23–95)	172/178	172/181	191	104	1/2/3/6	27	114
						Sofosbuvir/ velpatasvir plus ribavirin	67 (46–88)	9/9	9/10					
Taneja et al. (69)	2021	2018.09–2021.01	India	–	Real-life prospective study	Sofosbuvir/ velpatasvir	42.8 ± 14.6	49/51	49/51	51	41	1/3/4	10	51

SVR, sustained virologic response; TN, total number; OBV/PTV/R, ombitasvir/paritaprevir/ritonavir; DSV, dasabuvir; RBV, ribavirin; age (range), mean ± SD or median with IQR; dose adjustment, sofosbuvir dose reduction; ITT, intention to treat; mITT, modified intention to treat.

### Efficacy

#### Overall SVR

**Figure 3:**
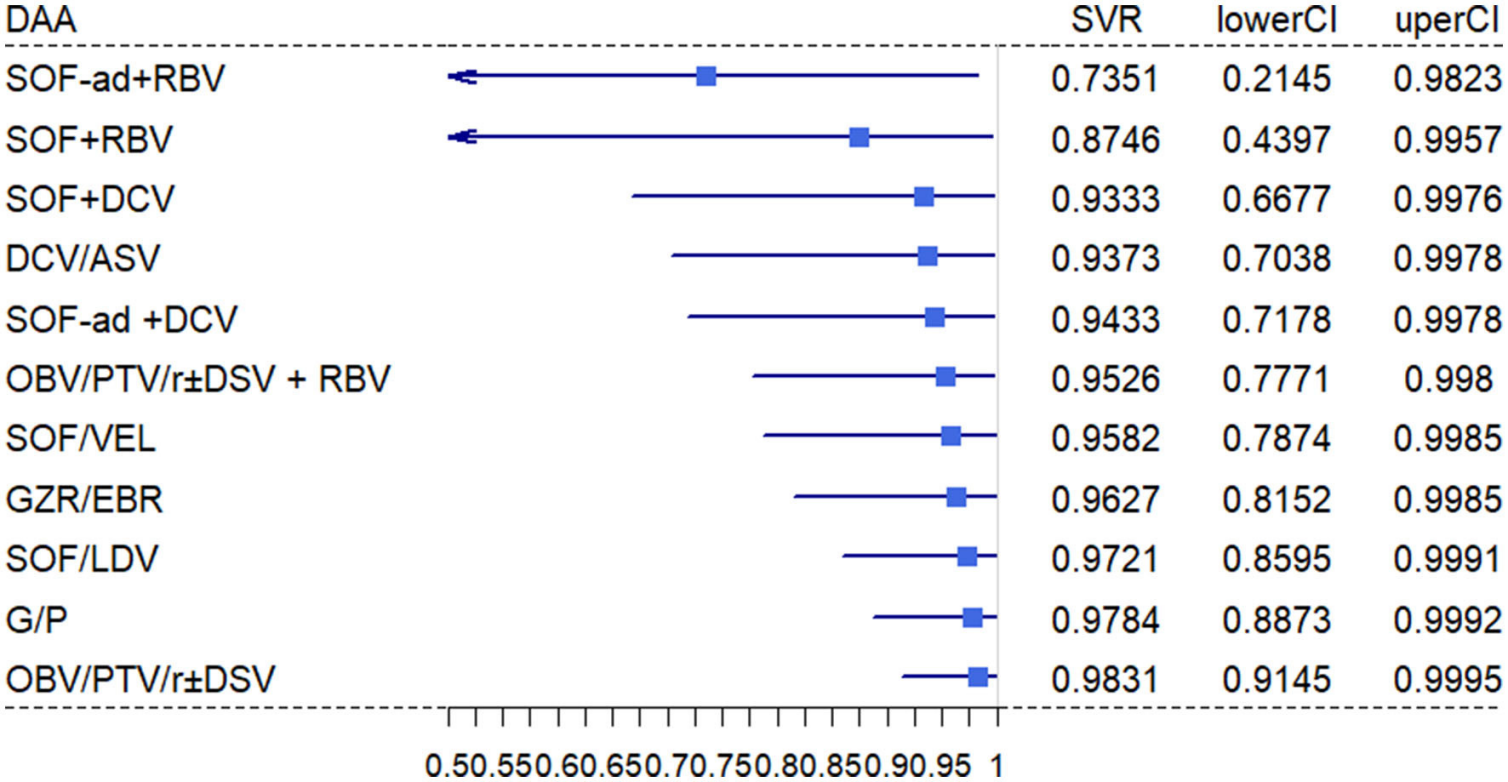
Estimated SVR rates per regimen. The mean estimated probability on SVR per regimen with 95% CI. The SVR rates are estimated for patients with EDSR.

Our network connected 11 all-oral DAA regimens to estimate pooled SVR in HCV-infected ESRD patients. The primary efficacy according to mITT analyses found the top five DAAs, followed by OBV/PTV/R ± DSV (98.31%; 95% CI, 91.45%−99.95%), G/P (97.84%; 95% CI, 88.73%−99.92%), SOF/LDV (97.21%; 95% CI, 85.95%−99.91%), GZR/EBR (96.27%; 95% CI, 81.52%−99.85%), and SOF/VEL (95.82%; 95% CI, 78.74%−99.85%; Figure 3). Using ITT analysis, the OBV/PTV/R ± DSV, G/P, and SOF/LDV were still the most effective DAAs with SVR >95% (Supplementary Figure 1).

#### Subgroup analyses by genotype of HCV

The studies on DAA's efficacy for HCV-infected ESRD patients have differences in genotypes. A total of 12 studies involving 694 patients with genotype 1a were used to estimate a pooled SVR of 97.5% (95% CI, 95.8%−99.2%). Among the included DAA regimens, OBV/PTV/R ± DSV (100%; 95% CI, 90.7%−100.0%), SOF/LDV (100.0%; 95% CI, 84.6%−100.0%), GZR/EBR (96.4%; 95% CI, 94.8%−98.0%), SOF + RBV (100%; 95% CI, 80.5%−100.0%), OBV/PTV/R ± DSV + RBV (97.4%; 95% CI, 92.8%−100.0%), and G/P (100.0%; 95% CI, 95.3%−100%) had high SVR rates more than 95% (Supplementary Figure 2). On genotype 1b, 22 studies involving 739 patients estimated a pooled SVR of 99.6% (95% CI, 98.0%−100%). All the DAAs showed excellent efficacy with high SVR rates over 95%: G/P (100.0%; 95% CI, 99.0%−100.0%), OBV/PTV/R ± DSV (99.8%; 95% CI, 97.0%−100.0%), OBV/PTV/R ± DSV + RBV (100.0%; 95% CI, 95.2%−100.0%), DCV + ASV (99.2%; 95% CI, 91.2%−100.0%), and GZR + EBR (96.6%; 95% CI, 93.2%−99.4%; Supplementary Figure 3).

Five G/P studies and one SOF + RBV study provided the data used for genotype 2 HCV-infected ESRD patients. The G/P regimen showed a pooled SVR of 98.9% (95% CI, 96.7%−100%). For patients with genotype 3, 13 studies contained G/P, SOF + RBV, SOF/DCV, SOF-ad/DCV, SOF/LDV, and SOF/VEL regimens reported that treatment effects in ESRD patients, with an overall SVR rate of 98.1% (95% CI, 94.7%−100%). In most of the supported studies, SOF/DCV (98.7%; 95% CI, 93.0%−100.0%) exhibited particularly good performance; even with a reduction in the dose of SOF combined with DCV, the SVR rate was still over 98% (98.4%; 95% CI, 93.5%−100.0%). For genotype 4 patients with ESRD, OBV/PTV/R and OBV/PTV/R + RBV were the most studied regimens. The overall SVR rate was 98.1% (95% CI, 94.4%−99.9%), showing an outstanding effect (Supplementary Figure 4).

**Figure 4:**
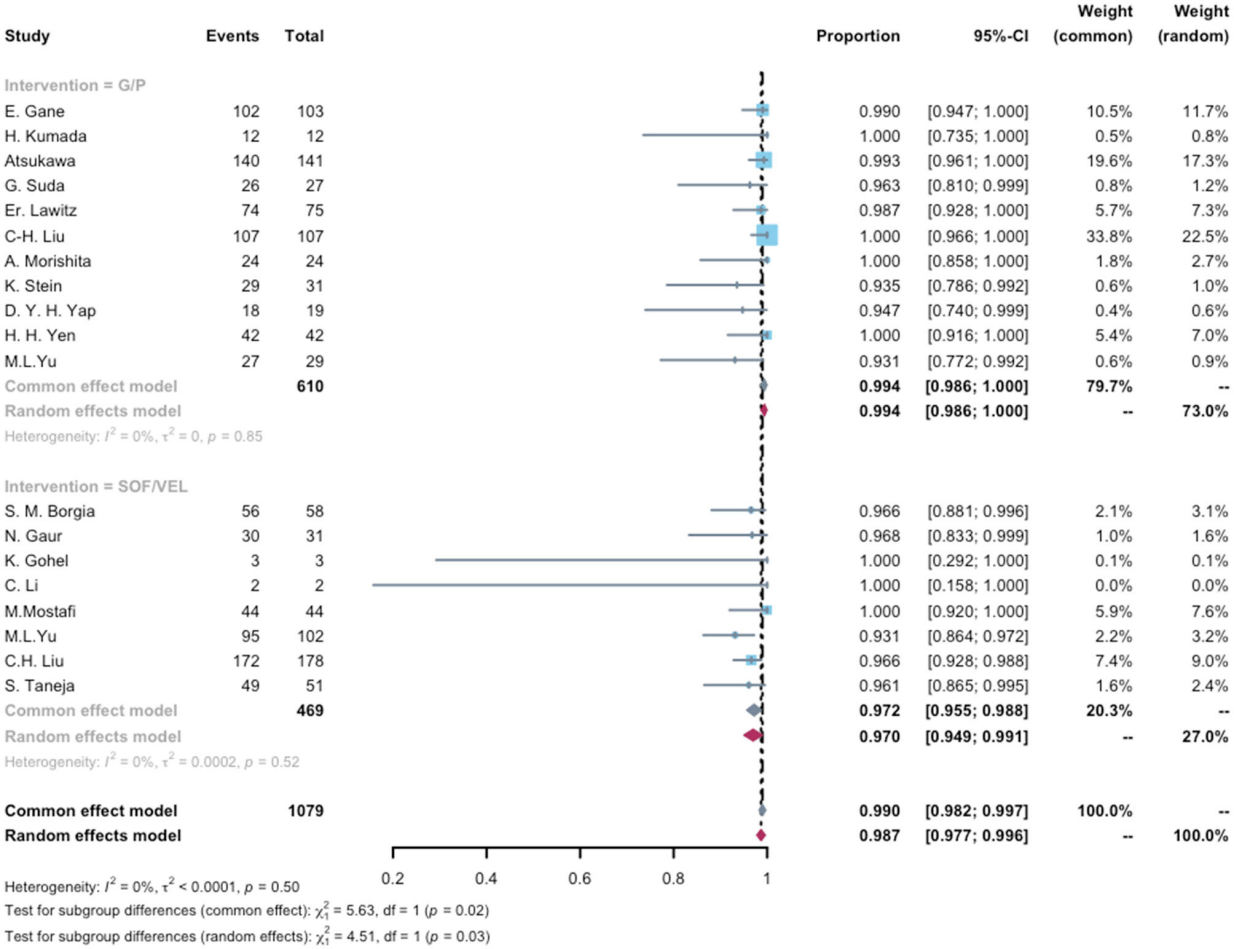
Compared SVR rates of pan-genotypic DAAs (G/P and SOL/VEL) regimens in end-stage renal disease patients with HCV infection. The mean pooled advent events per regimen with 95% CI. G/P, SOF, Sofosbuvir; VEL, Velpatasvir.

As the recommended pan-genotypic DAA therapy, G/P and SOL/VEL regimens used in HCV-infected ESRD patients included 19 studies for analysis. The G/P regimen showed a pooled SVR of 99.4% (95% CI, 98.6%−100%), and SOL/VEL had a suboptimal result with a pooled SVR of 97.0% (95% CI, 94.9%−99.1%; Figure 4).

#### DAA therapy subgroups analyses by grade of ESRD

Overall, 35 studies were identified to evaluate the efficacy of DAAs in HCV-infected patients with CKD5 or hemodialysis. Results show the overall SVR to be 97.5% (95% CI, 96.7%−98.4%). We also analyzed the efficacy of DAA in CHC patients with CKD4, with the overall SVR being 99.4% (95% CI, 97.4%−100.0%). Based on the most heavily weighted studies and patients, GZR/EBR and G/P had the highest SVR rates, almost 100%. Further comparison based on 10 studies involving GZR/EBR, G/P, OBV/PTV/R plus DSV or SOF-based regimens showed that the OR of achieving SVR in CKD4 vs. CKD5 was 0.75 (95% CI, 0.31–1.84) without significant difference (Supplementary Figure 5).

#### Subgroup analyses by cirrhosis

In this analysis, the included regimens of G/P, SOF/DCV, and OBV/PTV/R ± DSV ± RBV were included in the regimens for the majority of cirrhotic patients, with a high SVR of almost 100%. The OR of achieving SVR in cirrhotic compared to non-cirrhotic patients was 0.31 (95% CI, 0.14–0.69). No heterogeneity (*I*_2_ = 0) was found among these studies (Supplementary Figure 6).

### Safety

AEs were common in HCV-infected ESRD patients treated with DAAs. It was estimated that ~59.9% (95% CI, 50.3%−69.5%) of patients would experience at least one AE during the course, GZR/EBR (38.2%; 95% CI, 6.7%−69.7%), OBV/PTV/R (35.5%; 95% CI, 18.6%−52.3%), and G/P (49.7%; 95% CI, 33.7%−65.6%) had much lower AE rates than other DAA regimens (Figure 4). The DAA regimens without RBV or SOF had the lowest AE rate (49.9%; 95% CI, 38.4%−61.5%) in HCV-infected ESRD patients, whereas regimens with RBV or/and SOF could raise the AE rate to 63.3%−95.8% (Supplementary Figure 7).

The primary AEs included anemia (44.4%; 95% CI, 32.8%−56.0%), fatigue/asthenia (18.2%; 95% CI, 11.2%−25.2%), headache (12.2%; 95% CI, 4.9%−19.5%), diarrhea (6.2%; 95% CI, 3.1%−9.2%), nausea (9.3%; 95% CI, 6.1%−12.4%), insomnia (7.0%; 95% CI, 4.1%−9.9%), and dizziness (5.6%; 95% CI, 1.2%−10.1%; Supplementary Figures 8A, B). Anemia was the most common complication in ESRD patients with DAAs for HCV treatment. Further analyses showed that the incidence rate of hemoglobin ≤ 100 g/L was 41.2% (95% CI, 29.6%−52.7%), while the rate in none-RBV-containing regimens was 26.5% (95% CI, 17.7%−35.3%) vs. 63.0% (95% CI, 49.0%−77.1%) in RBV-containing regimens. The pooled incidence rate of severe anemia with hemoglobin ≤ 80 g/L was 5.40% (95% CI, 2.7%−8.2%), which was higher in regimens with RBV (8.7%; 95% CI, 0.7%−16.7%) than in regimens without RBV (4.7%; 95% CI, 2.2%−7.2%; Supplementary Figure 9).

Discontinuation of treatment and death were also the most important safety indicators for all-oral DAAs treatments. These were rarely reported in the included studies. The estimated pooled incidence rates of discontinuation of treatment and death were 0.8% (95% CI, 0.3%−1.3%) and 0.4% (95% CI, 0%−0.8%), respectively. However, the overall SAE incidence was 8.4% (95% CI, 5.2%−11.7%) by meta-analysis estimation. The pooled SAE and mortality rates reported in OBV/PTV/R plus DSV + RBV treatment were 24.8% (95% CI, 5.9%−43.7%) and 4% (95% CI, 0%−15.6%), respectively, and were highest among the treatment regimens. The top three drugs with the highest discontinuation rates were SOF/VEL + RBV (10%, 95% CI, 0%−28.6%), OBV/PTV/R (7.1%, 95% CI, 0%−21.1%), and SOF + RBV (6.2%, 95% CI, 0%−24.3%; Supplementary Figure 10).

## Discussion

This systematic review and network meta-analysis aimed to establish a hierarchy of available treatment regimens for HCV infection among patients with ESRD. To the best of our knowledge, this is the most comprehensive overview of the available efficacy and safety data for oral DAA regimens, and the main findings can be summarized below.

The key finding was that the OBV/PTV/R ± DSV regimen achieved the highest efficacy in HCV-infected ESRD patients, and similar estimated SVR rates could be achieved using the GP regimen. In addition, SOF/LDV, GZR/EBR, and SOF/VEL had only 1%−2% lower estimated SVR rates and remained alternative options for treatment.

We established the unprofitable value of Ribavirin, regardless of the difference in DAAs. The RBV did not improve the SVR of OBV/PTV/R ± DSV regimens in HCV-infected ESRD patients. The SOF-RBV and SOF-ad-RBV have the lowest SVR rates, poor performance, and should be considered obsolete.

Identifying certain genotypes before initiating therapy remains useful and may be required when drugs permit limitations or optimize treatment regimens. This study also gave priority to the selection of DAAs based on different genotypes. For HCV genotype 1a patients, in addition to OBV/PTV/R ± DSV and G/P as the optimal selection, two other combinations with SVR rates over 95% (SOF/LDV and GZR/EBR) would be recommended. Genotype 1b patients achieved excellent efficacy both in OBV/PTV/R ± DSV, GZR/EBR, and DCV/ASV, with SVR rates of approximately 99%. The G/P regimen would be the optimal solution for HCV genotype 2 ESRD patients based on the most evidence. As a relatively easy-to-treat type, HCV genotype 4 ESRD patients could achieve a higher SVR rate through OBV/PTV/R, and there were reasonable reasons to believe in the efficacy of other regimens. However, HCV genotype 3 is considered the most hard-to-treat type due to the increased incidence of cirrhosis that may reduce the SVR rate. In this meta-analysis, the SVR rate of SOF/DCV was close to 98%, and even a dose reduction of SOF combined with DCV also achieved an SVR rate of more than 95%. Thus, SOF/DCV would be a priority for genotype 3 ESRD patients, which was consistent with a previous network meta-analysis of optimal DAAs for HCV genotype 3 infection (73). As a pan-genotypic HCV drug regimen, the G/P regimen can be used to treat individuals without identifying their HCV genotype and subtype (74). In ESRD patients, G/P also showed a therapeutic superiority in all genotype subgroup analyses. The 2020 EASL (74) and 2019 AASLD (75) treatment guidelines now suggest two main regimens for G/P and SOF/VEL with pan-genotypic antiviral activity to simplify the treatment algorithm. The tolerability and effectiveness of pan-genotypic DAAs in ESRD are still unclear. By comparing those DAAs, we found the SVR of G/P was close to perfect and slightly better than SOL/VEL.

Subgroup analyses of cirrhosis suggested that ESRD patients with cirrhosis were 69% less likely to achieve SVR than those without cirrhosis. However, the influence of cirrhosis on efficacy was limited to ESRD patients using G/P and SOF/DCV regimens. Regardless of glomerular filtration rate, GZR/EBR or G/P used in ESRD patients both showed significant and comparable efficacy between CKD4 and CKD5 patients. When HCV-infected ESRD patients undergo hemodialysis, the OBV/PTV/R plus DSV and GP regimens are preferentially recommended due to the excellent efficacy of SVR, which exceeds 95%, which is much higher than other regimens. In addition, SOF/LDV, SOF/VEL, and GZR/EBR would also be good substitutes for hemodialytic patients as their SVR rates were around 94%.

A safety evaluation would identify incidences of AEs, discontinuation of treatment, and death in ESRD patients with different DAA regimens. AEs with the DAA regimens were common in ESRD patients, with an incidence of up to 59.9%. In contrast, the pooled occurrence rates of SAEs, discontinuation of treatment, and death were relatively much lower, at 8.4%, 0.8%, and 0.4%, respectively, among ESRD patients receiving DAA regimens. DAA regimens without SOF or RBV had a lower risk of common AEs.

Anemia was the most common of the reported AEs, with a pooled prevalence of 44.4%. Since anemia is a common complication of CKD, we further explored the side effects of RBV on anemia in ESRD patients. The results suggested that more than one-third of ESRD patients treated with DAAs would experience anemia, and the RBV-containing regimens increased the incidence of anemia by 30% compared with the RBV-free regimens. Moreover, anemia exacerbation was more common in patients on RBV-containing regimens. The probabilities of SAEs and deaths were much higher with OBV/PTV/R plus DSV + RBV and SOF + RBV regimens, which should be taken seriously. In addition, the SOF + RBV regimens had the highest discontinuation rate. Therefore, the use of RBV in the antiviral protocol for HCV-infected ESRD patients should be avoided. The SOF-based regimens like SOF/LDV and SOF/DCV both showed satisfactory safety profiles, which further confirmed their applicability in HCV-infected ESRD patients. The GP also had a solid safety profile with a 10% reduction in SAE rate and a 3% reduction in discontinuation rate, which had an extremely low risk of death as low as 0.1% in ESRD patients.

However, several limitations should be expounded and warrant further discussion. First, differences in the details of the study design resulted in significant heterogeneity among the included studies, which may compromise comparability. In response to this, we controlled for and explored sources of heterogeneity by choosing a random-effects model rather than a fixed-effects model for the analyses and completing subgroup analyses by genotype, cirrhosis, and ESRD class. Second, most publications reported SVR as the major outcome, with a brief accompanying safety assessment. Due to the small number of studies, it was not possible to compare SOF-based regimens with SOF-free regimens. Future studies will require detailed comparisons of safety among different DAAs. Third, the efficacy class of DAA regimens for specific HCV genotypes could not be determined, and the estimated pooled data for some DAA regimens related to specific genotypes could not be extracted separately due to the small number of patients. Fourth, we were unable to formally assess publication bias because the studies per regimen ranged from 1 to 11. Fifth, the efficacy in patients with ESRD and decompensated cirrhosis could not be explored due to the paucity of data. Sixth, the limitation of our network meta-analysis was the risk of conceptual heterogeneity, reflecting differences between trials that may impair comparability. We used several strategies to target heterogeneity: (1) we used a random-effects model (by including a study effect in our model); (2) we split the analyses for patients according to genotype, cirrhosis, and CKD grade; and (3) we performed analyses between mITT study and ITT study to increase homogeneity, which showed similar results. Moreover, SVR is an objective outcome that decreases the risk of heterogeneity. Nonetheless, we do not expect publication bias as the HCV field is rapidly evolving.

## Conclusion

The G/P would be recommended as the best option for the treatment of pan-genotypic HCV-infected ESRD patients due to its highest efficacy and safety; the SOF/VEL would be a suboptimal option. SOF/DCV had an advantage in the treatment of genotype 3 HCV patients. SOF-based DAA regimens had satisfactory safety profiles in HCV-infected ESRD patients; meanwhile, RBV should be counted out from HCV antiviral regimens in ESRD patients.

## Data availability statement

The original contributions presented in the study are included in the article/Supplementary material, further inquiries can be directed to the corresponding author.

## Author contributions

YYZ and YHX carried out the literature screening. YHX and RCC made the assessment and data extraction. YXZ and RCC wrote the article and prepared the figures and tables. YXZ designed the research and made the data analysis. YZX and XLW reviewed all the data, code, analysis, and results. All authors contributed to the article and approved the submitted version.

## References

[1] Nguyen DB Bixler D Patel PR. Transmission of hepatitis C virus in the dialysis setting and strategies for its prevention. Semin Dial. (2019) 32:127–34. 10.1111/sdi.12761 30569604 PMC6411055

[2] Carrat F Fontaine H Dorival C Simony M Diallo A Hezode C . Clinical outcomes in patients with chronic hepatitis C after direct-acting antiviral treatment: a prospective cohort study. Lancet. (2019) 393:1453–64. 10.1016/S0140-6736(18)32111-1 30765123

[3] Fabrizi F Donato FM Messa P. Association between hepatitis C virus and chronic kidney disease: a systematic review and meta-analysis. Ann Hepatol. (2018) 17:364–91. 10.5604/01.3001.0011.7382 29735788

[4] Gordon CE Berenguer MC Doss W Fabrizi F Izopet J Jha V . Prevention, diagnosis, evaluation, and treatment of hepatitis C virus infection in chronic kidney disease: synopsis of the kidney disease: improving global outcomes 2018 clinical practice guideline. Ann Intern Med. (2019) 171:496–504. 10.7326/M19-1539 31546256

[5] Jadoul M Berenguer MC Doss W Fabrizi F Izopet J Jha V . Executive summary of the 2018 KDIGO hepatitis C in CKD guideline: welcoming advances in evaluation and management. Kidney Int. (2018) 94:663–73. 10.1016/j.kint.2018.06.011 30243313

[6] Hutton B Salanti G Caldwell DM Chaimani A Schmid CH Cameron C . The PRISMA extension statement for reporting of systematic reviews incorporating network meta-analyses of health care interventions: checklist and explanations. Ann Intern Med. (2015) 162:777–84. 10.7326/M14-2385 26030634

[7] Slim K Nini E Forestier D Kwiatkowski F Panis Y Chipponi J. Methodological index for non-randomized studies (minors): development and validation of a new instrument. ANZ J Surg. (2003) 73:712–6. 10.1046/j.1445-2197.2003.02748.x 12956787

[8] Wells GA Shea B O'Connell D Peterson J Welch V Losos M . The Newcastle-Ottawa Scale (NOS) for Assessing the Quality of Nonrandomised Studies in Meta-Analyses. (2011). Available online at: https://www.ohri.ca/programs/clinical_epidemiology/oxford.asp (accessed August 10, 2023).

[9] Chronic Hepatitis C Virus Infection: Developing Direct-Acting Antiviral Drugs for Treatment: Guidance for Industry (2017). Available online at: https://www.fda.gov/regulatory-information/search-fda-guidance-documents/chronic-hepatitis-c-virus-infection-developing-direct-acting-antiviral-drugs-treatment-guidance

[10] Dias S Welton NJ Sutton AJ Ades AE. Evidence synthesis for decision making 5: the baseline natural history model. Med Decis Making. (2013) 33:657–70. 10.1177/0272989X13485155 23804509 PMC3704201

[11] Roth D Nelson DR Bruchfeld A Liapakis A Silva M Monsour H . Grazoprevir plus elbasvir in treatment-naive and treatment-experienced patients with hepatitis C virus genotype 1 infection and stage 4-5 chronic kidney disease (the C-SURFER study): a combination phase 3 study. Lancet. (2015) 386:1537–45. 10.1016/S0140-6736(15)00349-9 26456905

[12] Kawakami Y Imamura M Ikeda H Suzuki M Arataki K Moriishi M . Pharmacokinetics, efficacy and safety of daclatasvir plus asunaprevir in dialysis patients with chronic hepatitis C: pilot study. J Viral Hepat. (2016) 23:850–6. 10.1111/jvh.12553 27346670

[13] Miyazaki R Miyagi K. Effect and safety of daclatasvir-asunaprevir combination therapy for chronic hepatitis C virus genotype 1b-infected patients on hemodialysis. Ther Apher Dial. (2016) 20:462–7. 10.1111/1744-9987.12407 27098678

[14] Pockros PJ Reddy KR Mantry PS Cohen E Bennett M Sulkowski MS . Efficacy of direct-acting antiviral combination for patients with hepatitis C virus genotype 1 infection and severe renal impairment or end-stage renal disease. Gastroenterology. (2016) 150:1590–8. 10.1053/j.gastro.2016.02.078 26976799

[15] Suda G Kudo M Nagasaka A Furuya K Yamamoto Y Kobayashi T . Efficacy and safety of daclatasvir and asunaprevir combination therapy in chronic hemodialysis patients with chronic hepatitis C. J Gastroenterol. (2016) 51:733–40. 10.1007/s00535-016-1162-8 26768604

[16] Toyoda H Kumada T Tada T Takaguchi K Ishikawa T Tsuji K . Safety and efficacy of dual direct-acting antiviral therapy (daclatasvir and asunaprevir) for chronic hepatitis C virus genotype 1 infection in patients on hemodialysis. J Gastroenterol. (2016) 51:741–7. 10.1007/s00535-016-1174-4 26872889

[17] Abad S Vega A Hernandez E Merida E de Sequera P Albalate M . Universal sustained viral response to the combination of ombitasvir/paritaprevir/ritonavir and dasabuvir with/without ribavirin in patients on hemodialysis infected with hepatitis C virus genotypes 1 and 4. Am J Nephrol. (2017) 45:267–72. 10.1159/000454819 28166520

[18] Agarwal SK Bagchi S Yadav RK. Hemodialysis patients treated for hepatitis C using a sofosbuvir-based regimen. Kidney Int Rep. (2017) 2:831–5. 10.1016/j.ekir.2017.04.003 29270489 PMC5733818

[19] Atsukawa M Tsubota A Koushima Y Ikegami T Watanabe K Shimada N . Efficacy and safety of ombitasvir/paritaprevir/ritonavir in dialysis patients with genotype 1b chronic hepatitis C. Hepatol Res. (2017) 47:1429–37. 10.1111/hepr.12910 28457003

[20] Gane E Lawitz E Pugatch D Papatheodoridis G Bräu N Brown A . Glecaprevir and pibrentasvir in patients with HCV and severe renal impairment [clinical trial, phase III multicenter study]. N Engl J Med. (2017) 377:1448–55. 10.1056/NEJMoa1704053 29020583

[21] Morisawa N Koshima Y Kuriyama S Matsuyama M Hayashi N Satoh JI . Effectiveness of a fixed combination formula of ombitasvir/paritaprevir/ritonavir for hepatitis C virus infection in patients on maintenance haemodialysis. Nephrology. (2017) 22:562–5. 10.1111/nep.13011 28621007

[22] Munoz-Gomez R Rincon D Ahumada A Hernandez E Devesa MJ Izquierdo S . Therapy with ombitasvir/paritaprevir/ritonavir plus dasabuvir is effective and safe for the treatment of genotypes 1 and 4 hepatitis C virus (HCV) infection in patients with severe renal impairment: a multicentre experience. J Viral Hepat. (2017) 24:464–71. 10.1111/jvh.12664 27976490

[23] Otsuka T Kawaguchi Y Mizuta T Ide Y Koga F Kumagai T . Asunaprevir and daclatasvir in hemodialysis patients with chronic hepatitis C virus genotype 1b infection. Jgh Open. (2017) 1:148–52. 10.1002/jgh3.12026 30483552 PMC6207006

[24] Sperl J Frankova S Kreidlova M Merta D Tothova M Spicak J. Combination of sofosbuvir and daclatasvir in the treatment of genotype 3 chronic hepatitis C virus infection in patients on maintenance hemodialysis. Ther Clin Risk Manag. 2017 (2017) 13:733–8. 10.2147/TCRM.S133983 28790832 PMC5488764

[25] Alric L Ollivier-Hourmand I Berard E Hillaire S Guillaume M Vallet-Pichard A . Grazoprevir plus elbasvir in HCV genotype-1 or-4 infected patients with stage 4/5 severe chronic kidney disease is safe and effective. Kidney Int. (2018) 94:206–13. 10.1016/j.kint.2018.02.019 29735308

[26] Butt AA Ren Y Puenpatom A Arduino JM Kumar R Abou-Samra AB. Effectiveness, treatment completion and safety of sofosbuvir/ledipasvir and paritaprevir/ritonavir/ombitasvir + dasabuvir in patients with chronic kidney disease: an ERCHIVES study [research support, Non-US Gov't]. Aliment Pharmacol Ther. (2018) 48:35–43. 10.1111/apt.14799 29797514

[27] Fujii H Kimura H Kurosaki M Hasebe C Akahane T Yagisawa H . Efficacy of daclatasvir plus asunaprevir in patients with hepatitis C virus infection undergoing and not undergoing hemodialysis. Hepatol Res. (2018) 48:746–56. 10.1111/hepr.13070 29480939

[28] Gupta A Arora P Jain P. Sofosbuvir based regimen in management of hepatitis c for patients with end stage renal disease on hemodialysis: a single center experience from India. J Clin Exp Hepatol. (2018) 8:116–20. 10.1016/j.jceh.2017.10.001 29892172 PMC5992300

[29] Kumada H Watanabe T Suzuki F Ikeda K Sato K Toyoda H . Efficacy and safety of glecaprevir/pibrentasvir in HCV-infected Japanese patients with prior DAA experience, severe renal impairment, or genotype 3 infection [clinical trial, phase III multicenter study]. J Gastroenterol. (2018) 53:566–75. 10.1007/s00535-017-1396-0 29052790 PMC5866827

[30] Manoj K Nayak SL Gupta E Kataria A Sarin SK. Generic sofosbuvir-based direct-acting antivirals in hepatitis C virus-infected patients with chronic kidney disease. Liver Int. (2018) 38:2137–48. 10.1111/liv.13863 29676846

[31] Ogawa E Furusyo N Azuma K Nakamuta M Nomura H Dohmen K . Elbasvir plus grazoprevir for patients with chronic hepatitis C genotype 1: a multicenter, real-world cohort study focusing on chronic kidney disease. Antiviral Res. (2018) 159:143–52. 10.1016/j.antiviral.2018.10.003 30300717

[32] Sanai FM Alghamdi AS Afghani AA Alswat K AlZanbagi A Alghamdi MN . High efficacy of ombitasvir/paritaprevir/ritonavir plus dasabuvir in hepatitis C genotypes 4 and 1-infected patients with severe chronic kidney disease. Liver Int. (2018) 38:1395–401. 10.1111/liv.13674 29288514

[33] Sperl J Kreidlova M Merta D Chmelova K Senkerikova R Frankova S. Paritaprevir/ritonavir/ombitasvir plus dasabuvir regimen in the treatment of genotype 1 chronic hepatitis c infection in patients with severe renal impairment and end-stage renal disease: a real-life cohort. Kidney Blood Press Res. (2018) 43:594–605. 10.1159/000488965 29669332

[34] Suda G Furusyo N Toyoda H Kawakami Y Ikeda H Suzuki M . Daclatasvir and asunaprevir in hemodialysis patients with hepatitis C virus infection: a nationwide retrospective study in Japan. J Gastroenterol. (2018) 53:119–28. 10.1007/s00535-017-1353-y 28560477

[35] Taneja S Duseja A De A Mehta M Ramachandran R Kumar V . Low-dose sofosbuvir is safe and effective in treating chronic hepatitis c in patients with severe renal impairment or end-stage renal disease [observational study]. Dig Dis Sci. (2018) 63:1334–40. 10.1007/s10620-018-4979-6 29484572

[36] Atsukawa M Tsubota A Toyoda H Takaguchi K Kondo C Okubo T . Efficacy and safety of elbasvir/grazoprevir for Japanese patients with genotype 1b chronic hepatitis C complicated by chronic kidney disease, including those undergoing hemodialysis: a *post hoc* analysis of a multicenter study. J Gastroenterol Hepatol. (2019) 34:364–9. 10.1111/jgh.14447 30144366

[37] Atsukawa M Tsubota A Toyoda H Takaguchi K Nakamuta M Watanabe T . The efficacy and safety of glecaprevir plus pibrentasvir in 141 patients with severe renal impairment: a prospective, multicenter study. Aliment Pharmacol Ther. (2019) 49:1230–41. 10.1111/apt.15218 30873651

[38] Aydin NN Aksoy F Yavuz I Iskender S Yildirim AA Yildiz IE . Efficacy of direct-acting antivirals in hemodialysis patients with chronic hepatitis C: a real-life retrospective study. Viral Hepat J. (2019) 25:105–8. 10.4274/vhd.galenos.2019.2019.002529582685

[39] Borgia SM Dearden J Yoshida EM Shafran SD Brown A Ben-Ari Z . Sofosbuvir/velpatasvir for 12weeks in hepatitis C virus-infected patients with end-stage renal disease undergoing dialysis. J Hepatol. (2019) 71:660–5. 10.1016/j.jhep.2019.05.028 31195062

[40] Butt N Abbasi A Khan MA Ali M Mahesar GB Haleem F . Treatment outcomes for patients undergoing hemodialysis with chronic hepatitis C on the Sofosbuvir and daclatasvir regimen. Cureus. (2019) 11:e5702. 10.7759/cureus.5702 31720170 PMC6823026

[41] Cheema SUR Rehman MS Hussain G Cheema SS Gilani N. Efficacy and tolerability of sofosbuvir and daclatasvir for treatment of hepatitis C genotype 1 & 3 in patients undergoing hemodialysis- a prospective interventional clinical trial. BMC Nephrol. (2019) 20:438. 10.1186/s12882-019-1631-4 31779583 PMC6883698

[42] Elmowafy AY El Maghrabi HM Eldahshan KF Refaie AF Elbasiony MA Matter YE . Treatment of hepatitis C infection among Egyptian hemodialysis patients: the dream becomes a reality. Int Urol Nephrol. (2019) 51:1639–47. 10.1007/s11255-019-02246-7 31363959

[43] Goel A Bhadauria DS Kaul A Verma P Mehrotra M Gupta A . Daclatasvir and reduced-dose sofosbuvir: an effective and pangenotypic treatment for hepatitis C in patients with estimated glomerular filtration rate. Nephrology. (2019) 24:316–21. 10.1111/nep.13222 29327401

[44] Lawitz E Gane E Cohen E Vierling J Agarwal K Hassanein T . Efficacy and Safety of ombitasvir/paritaprevir/ritonavir in patients with hepatitis c virus genotype 1 or 4 infection and advanced kidney disease. Kidney Int Rep. (2019) 4:257–66. 10.1016/j.ekir.2018.10.003 30775622 PMC6365406

[45] Lee BS Song MJ Kwon JH Lee TH Jang JW Kim SH . Efficacy and safety of daclatasvir and asunaprevir in patients with hepatitis C virus genotype 1b infection on hemodialysis. Gut Liver. (2019) 13:191–6. 10.5009/gnl18240 30400729 PMC6430432

[46] Maduell F Belmar L Ugalde J Laguno M Martinez-Rebollar M Ojeda R . Elimination of hepatitis C virus infection from a hemodialysis unit and impact of treatment on the control of anemia. Gastroenterol Hepatol. (2019) 42:164–70. 10.1016/j.gastrohep.2018.07.015 30293914

[47] Mekky MA Abdel-Malek MO Osman HA Abdel-Aziz EM Hashim AKA Hetta HF . Efficacy of ombitasvir/paritaprevir/ritonavir/ribavirin in management of HCV genotype 4 and end-stage kidney disease. Clin Res Hepatol Gastroenterol. (2019) 43:82–7. 10.1016/j.clinre.2018.08.003 30166253

[48] Suda G Hasebe C Abe M Kurosaki M Itakura J Izumi N . Safety and efficacy of glecaprevir and pibrentasvir in Japanese hemodialysis patients with genotype 2 hepatitis C virus infection [multicenter study]. J Gastroenterol. (2019) 54:641–9. 10.1007/s00535-019-01556-y 30778716

[49] Tatar B Köse S Ergun NC Turken M Onlen Y Yilmaz Y . Response to direct-acting antiviral agents in chronic hepatitis C patients with end-stage renal disease: a clinical experience. Rev Assoc Med Bras. (2019) 65:1470–5. 10.1590/1806-9282.65.12.1470 31994628

[50] Yaraş S Üçbilek E Özdogan O Ateş F Altintaş E Sezgin O. Real-life results of treatment with ombitasvir, paritaprevir, dasabuvir, and ritonavir combination in patients with chronic renal failure infected with HCV in Turkey. Turk J Gastroenterol. (2019) 30:331–5. 10.5152/tjg.2018.18269 30666967 PMC6453652

[51] Abd-Elsalam S Abo-Amer YEE El-Abgeegy M Elshweikh SA Elsergany HF Ahmed R . Efficacy and safety of ombitasvir/paritaprevir/ ritonavir/ribavirin in management of Egyptian chronic hepatitis C virus patients with chronic kidney disease A real-life experience. Medicine. (2020) 99:e21972. 10.1097/MD.0000000000021972 33080669 PMC7572016

[52] Choi DT Puenpatom A Yu X Erickson KF Kanwal F El-Serag HB . Effectiveness of elbasvir/grazoprevir in patients with hepatitis C virus genotype 1 infection and chronic kidney disease in the United States veterans population. Antiviral Res. (2020) 174:104698. 10.1016/j.antiviral.2019.104698 31862503 PMC8724917

[53] Debnath P Chandnani S Rath P Nair S Pawar V Contractor Q. Combined NS5A & NS5B nucleotide inhibitor therapy for patients with chronic hepatitis c with stage 5 chronic kidney disease on hemodialysis. Arq Gastroenterol. (2020) 57:39–44. 10.1590/s0004-2803.202000000-08 32294734

[54] Eletreby R El-Serafy M Anees M Kasem G Salama M Elkhouly R . Sofosbuvir-containing regimens are safe and effective in the treatment of HCV patients with moderate to severe renal impairment. Liver Int. (2020) 40:797–805. 10.1111/liv.14299 31858694

[55] Gaur N Malhotra V Agrawal D Singh SK Beniwal P Sharma S . Sofosbuvir–velpatasvir fixed drug combination for the treatment of chronic hepatitis c infection in patients with end-stage renal disease and kidney transplantation. J Clin Exp Hepatol. (2020) 10:189–93. 10.1016/j.jceh.2019.10.004 32405174 PMC7212294

[56] Gohel K Borasadia P. Sofosbuvir-based HCV treatment in maintenance hemodialysis patients: a single-center study. Transplant Proc. (2020) 52:1684–6. 10.1016/j.transproceed.2020.02.136 32507712

[57] Lawitz E Landis CS Flamm SL Bonacini M Ortiz-Lasanta G Huang J . Sofosbuvir plus ribavirin and sofosbuvir plus ledipasvir in patients with genotype 1 or 3 hepatitis C virus and severe renal impairment: a multicentre, phase 2b, non-randomised, open-label study. Lancet Gastroenterol Hepatol. (2020) 5:918–26. 10.1016/S2468-1253(19)30417-0 32531259

[58] Li C Liang J Xiang H Chen H Tian J. Effectiveness of direct-acting antivirals in maintenance hemodialysis patients complicated with chronic hepatitis C. Medicine. (2020) 99:e23384. 10.1097/MD.0000000000023384 33235113 PMC7710190

[59] Liu C-H Peng C-Y Fang Y-J Kao W-Y Yang S-S Lin C-K . Elbasvir/grazoprevir for hepatitis C virus genotype 1b East-Asian patients receiving hemodialysis. Sci. Rep. (2020) 10:9180. 10.1038/s41598-020-66182-8 32513953 PMC7280513

[60] Liu C-H Yang S-S Peng C-Y Lin W-T Liu C-J Su T-H . Glecaprevir/pibrentasvir for patients with chronic hepatitis C virus infection and severe renal impairment. J Viral Hepat. (2020) 27:568–75. 10.1111/jvh.13265 31981264

[61] Morishita A Ogawa C Moriya A Tani J Yoneyama H Fujita K et al. Clinical outcomes of hepatitis C virus elimination using glecaprevir and pibrentasvir in hemodialysis patients: a multicenter study. Hepatol. Res. (2020) 50:557–64. 10.1111/hepr.13482 31883211

[62] Poustchi H Majd Jabbari S Merat S Sharifi A-H Shayesteh AA Shayesteh E . The combination of sofosbuvir and daclatasvir is effective and safe in treating patients with hepatitis C and severe renal impairment. J. Gastroenterol. Hepatol. (2020) 35:1590–4. 10.1111/jgh.14994 31994788

[63] Seo HY Seo MS Yoon SY Choi JW Ko SY. Full-dose sofosbuvir plus low-dose ribavirin for hepatitis c virus genotype 2-infected patients on hemodialysis. Korean J Intern Med. (2020) 35:559–65. 10.3904/kjim.2018.338 31064176 PMC7214360

[64] Stein K Stoehr A Klinker H Teuber G Naumann U John C . Hepatitis C therapy with grazoprevir/elbasvir and glecaprevir/pibrentasvir in patients with advanced chronic kidney disease: data from the German Hepatitis C-Registry (DHC-R). Eur J Gastroenterol Hepatol. (2020) 34:76–83. 10.1055/s-0040-1716035 32956186

[65] Yap DYH Liu KSH Hsu YC Wong GLH Tsai MC Chen CH . Use of glecaprevir/pibrentasvir in patients with chronic hepatitis C virus infection and severe renal impairment. Clin Mol Hepatol. (2020) 26:554–61. 10.3350/cmh.2020.0058 32854457 PMC7641551

[66] Yen HH Su PY Zeng YH Liu IL Huang SP Hsu YC . Glecaprevir-pibrentasvir for chronic hepatitis C: comparing treatment effect in patients with and without end-stage renal disease in a real-world setting. PLoS ONE. (2020) 15:e0237582. 10.1371/journal.pone.0237582 32790715 PMC7425913

[67] Cheng PN Chen CY Yu ML Lin CC Lin CY Peng CY . Elbasvir/grazoprevir is effective and tolerable for the treatment of HCV GT1-infected patients: a real world multicenter observatory study in Taiwan. J Microbiol Immunol Infect. (2021) 54:588–95. 10.1016/j.jmii.2020.05.004 32499107

[68] Liu C-H Chen C-Y Su W-W Tseng K-C Lo C-C Liu C-J . Sofosbuvir/velpatasvir with or without low-dose ribavirin for patients with chronic hepatitis C virus infection and severe renal impairment. Gut. (2021) 71:176–84. 10.1136/gutjnl-2020-323569 33408122

[69] Taneja S Duseja A Mehta M De A Verma N Premkumar M . Sofosbuvir and Velpatasvir combination is safe and effective in treating chronic hepatitis C in end-stage renal disease on maintenance haemodialysis. Liver Int. (2021) 41:705–9. 10.1111/liv.14685 33025685

[70] Lawitz E Flisiak R Abunimeh M Sise ME Park JY Kaskas M . Efficacy and safety of glecaprevir/pibrentasvir in renally impaired patients with chronic HCV infection. Liver Int. (2020) 40:1032–41. 10.1111/liv.14320 31821716

[71] Mostafi M Jabin M Chowdhury Z Khondoker MU Ali SM Tamanna R . The outcome of daclatasvir and low dose sofosbuvir therapy in end-stage renal disease patients with hepatitis C virus infection. Ukr J Nephrol Dial. (2020). 10.31450/ukrjnd.2(66).2020.01

[72] Yu ML Huang CF Wei YJ Lin WY Lin YH Hsu PY . Establishment of an outreach, grouping healthcare system to achieve microelimination of HCV for uremic patients in haemodialysis centres (ERASE-C). Gut. (2021) 70:2349–58. 10.1136/gutjnl-2020-323277 33303567

[73] Berden FA Aaldering BR Groenewoud H IntHout J Kievit W Drenth JP. Identification of the best direct-acting antiviral regimen for patients with hepatitis C virus genotype 3 infection: a systematic review and network meta-analysis. Clin Gastroenterol Hepatol. (2017) 15:349–59. 10.1016/j.cgh.2016.10.034 27840182

[74] European Association for the Study of the Liver. Electronic Address EEE, Clinical Practice Guidelines Panel C, representative EGB, Panel Members. EASL recommendations on treatment of hepatitis C: final update of the series(⋆). J Hepatol. (2020) 73:1170–18. 10.1016/j.jhep.2020.08.018 32956768

[75] Ghany MG Morgan TR. Hepatitis C guidance 2019 update: American association for the study of liver diseases-infectious diseases society of America recommendations for testing, managing, and treating hepatitis C Virus Infection. Hepatology. (2020) 71:686–721. 10.1002/hep.31060 31816111 PMC9710295

