# Supplementary material for: The efficacy and safety of direct-acting antiviral regimens for end-stage renal disease patients with HCV infection: a systematic review and network meta-analysis
Source: Front Public Health. 2023 Sep 29;11:1179531. doi: 10.3389/fpubh.2023.1179531 (PMC10570741; doi:10.3389/fpubh.2023.1179531)

Supplementary Material

The Efficacy and Safety of Direct-acting Antiviral regimens for end-stage renal disease patients with HCV infection: A Systematic review and Network meta-analysis

Ruo Chan Chen1 †, Yinghui Xiong1 †,Yanyang Zeng1, Xiaolei Wang2, Yinzong Xiao3, Yixiang Zheng 1*

*** Correspondence:** Yixiang Zheng, yxzheng@csu.edu.cn

Supplementary Figure 1. The primary efficacy according mITT analyses to estimate pooled SVR in HCV-infected ESRD patients

#
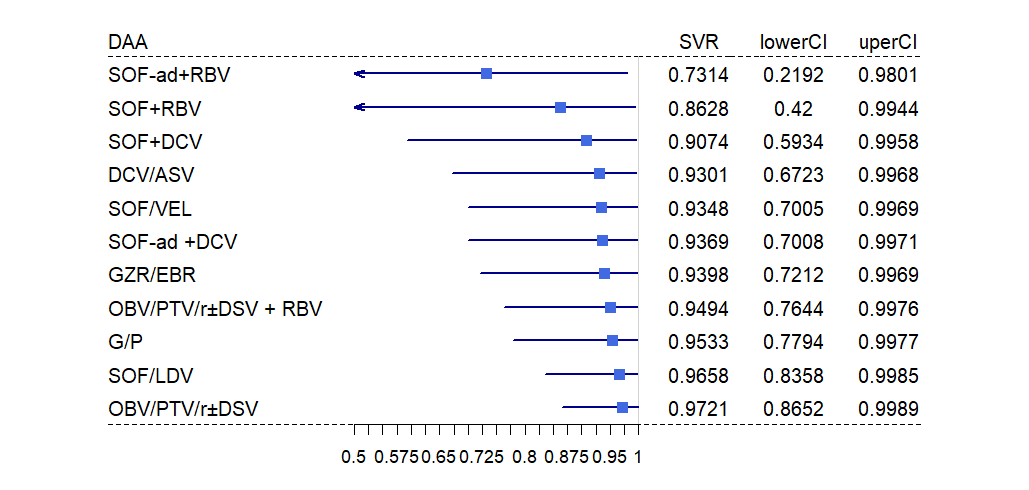

Supplement: Supplementary file 1 [file Data_Sheet_1.zip › Supplementary Figure 1.docx]
