# Supplementary material for: The efficacy and safety of direct-acting antiviral regimens for end-stage renal disease patients with HCV infection: a systematic review and network meta-analysis
Source: Front Public Health. 2023 Sep 29;11:1179531. doi: 10.3389/fpubh.2023.1179531 (PMC10570741; doi:10.3389/fpubh.2023.1179531)
Supplement: Supplementary file 1 [file Data_Sheet_1.zip › Supplementary Figure 6.docx]

***Supplementary Material***

**The** **Efficacy and** **Safety of Direct-acting Antiviral regimens for end-stage renal disease patients with HCV infection: A Systematic review and Network meta-analysis**

**Ruo Chan Chen1 †, Yinghui Xiong1 †,Yanyang Zeng1, Xiaolei Wang2, Yinzong Xiao3, Yixiang Zheng 1***

*** Correspondence:** Yixiang Zheng, yxzheng@csu.edu.cn

**Supplementary Figure 6**

**A**

**
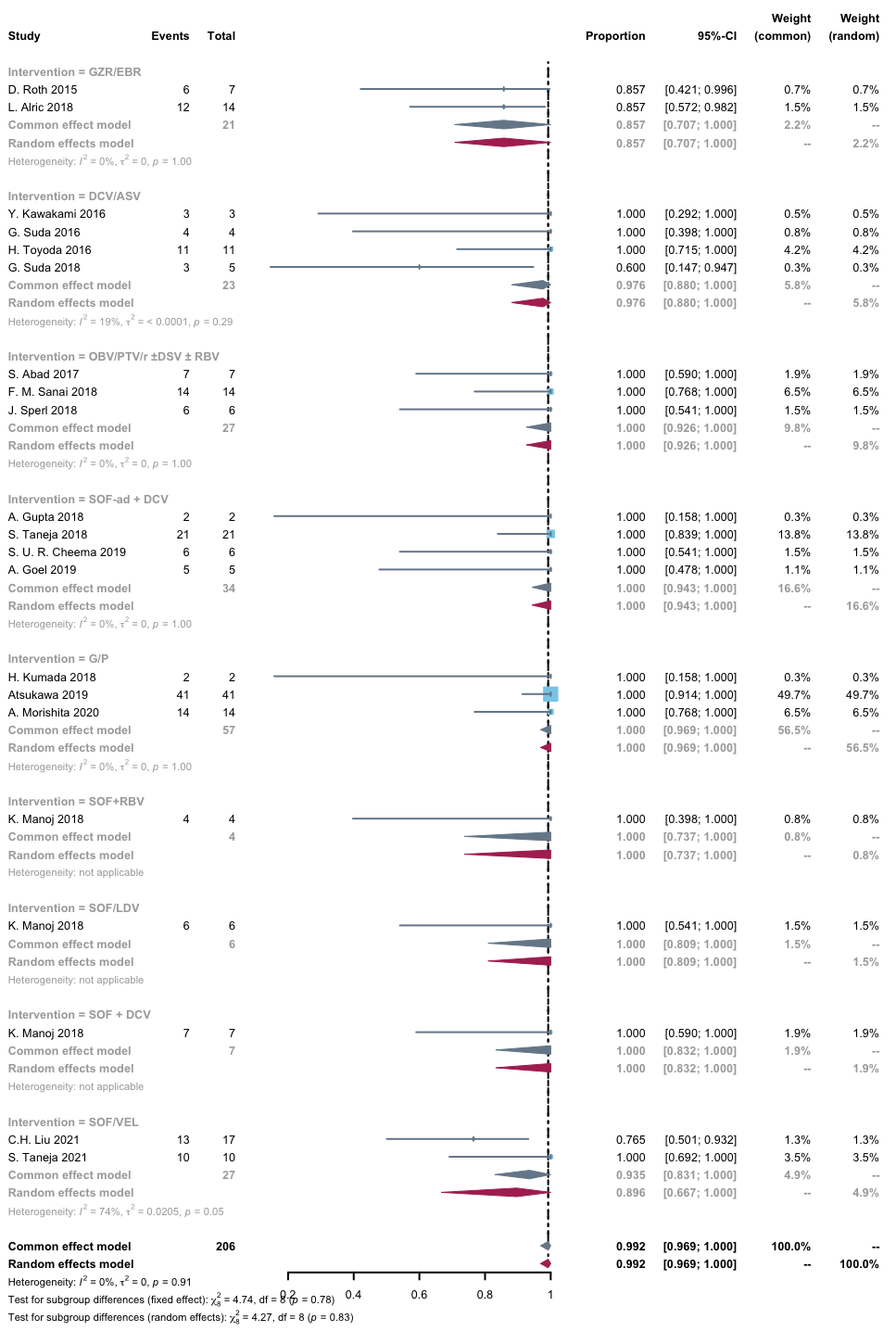
**

**B**

**
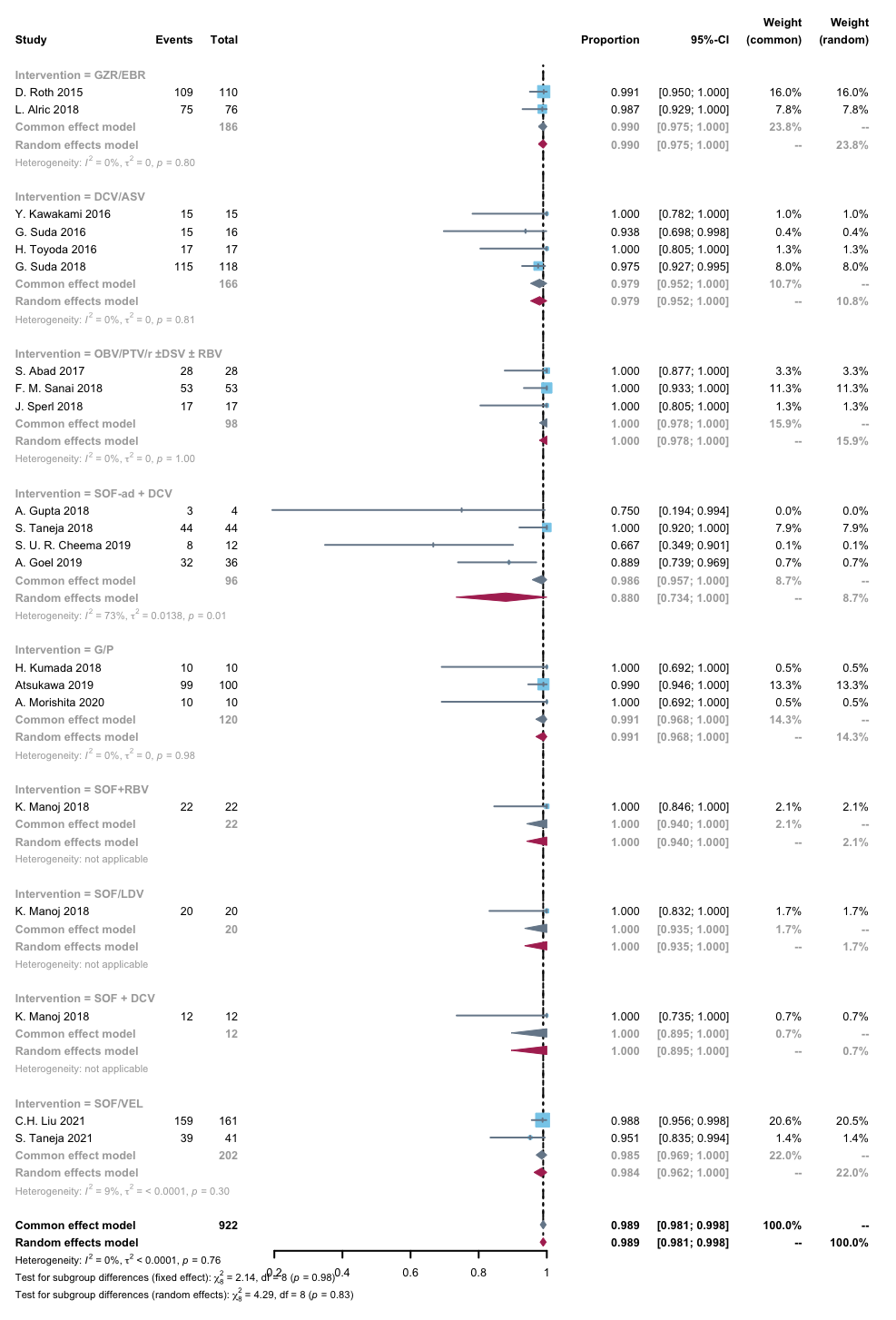
**

**C**

**
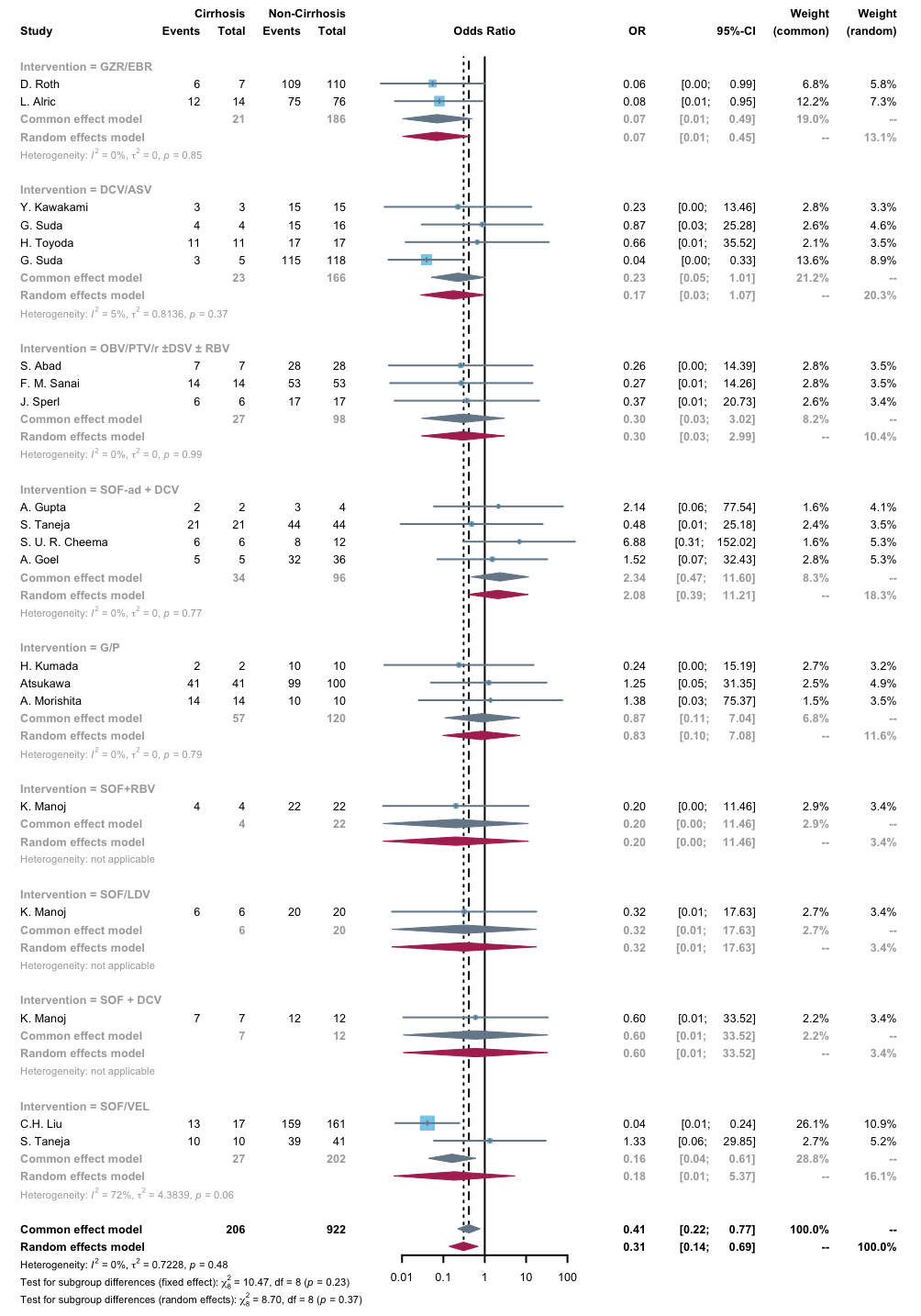
**

**Supplementary Figure 6.** Estimated pooled SVR of cirrhosis(A) and non-cirrhosis (B), the OR of achieving SVR in cirrhotic compared to non-cirrhotic patients.
