# Supplementary material for: The efficacy and safety of direct-acting antiviral regimens for end-stage renal disease patients with HCV infection: a systematic review and network meta-analysis
Source: Front Public Health. 2023 Sep 29;11:1179531. doi: 10.3389/fpubh.2023.1179531 (PMC10570741; doi:10.3389/fpubh.2023.1179531)
Supplement: Supplementary file 1 [file Data_Sheet_1.zip › Supplementary Figure 7.docx]

***Supplementary Material***

**The** **Efficacy and** **Safety of Direct-acting Antiviral regimens for end-stage renal disease patients with HCV infection: A Systematic review and Network meta-analysis**

**Ruo Chan Chen1 †, Yinghui Xiong1 †,Yanyang Zeng1, Xiaolei Wang2, Yinzong Xiao3, Yixiang Zheng 1***

*** Correspondence:** Yixiang Zheng, yxzheng@csu.edu.cn

**Supplementary Figure 7**

**
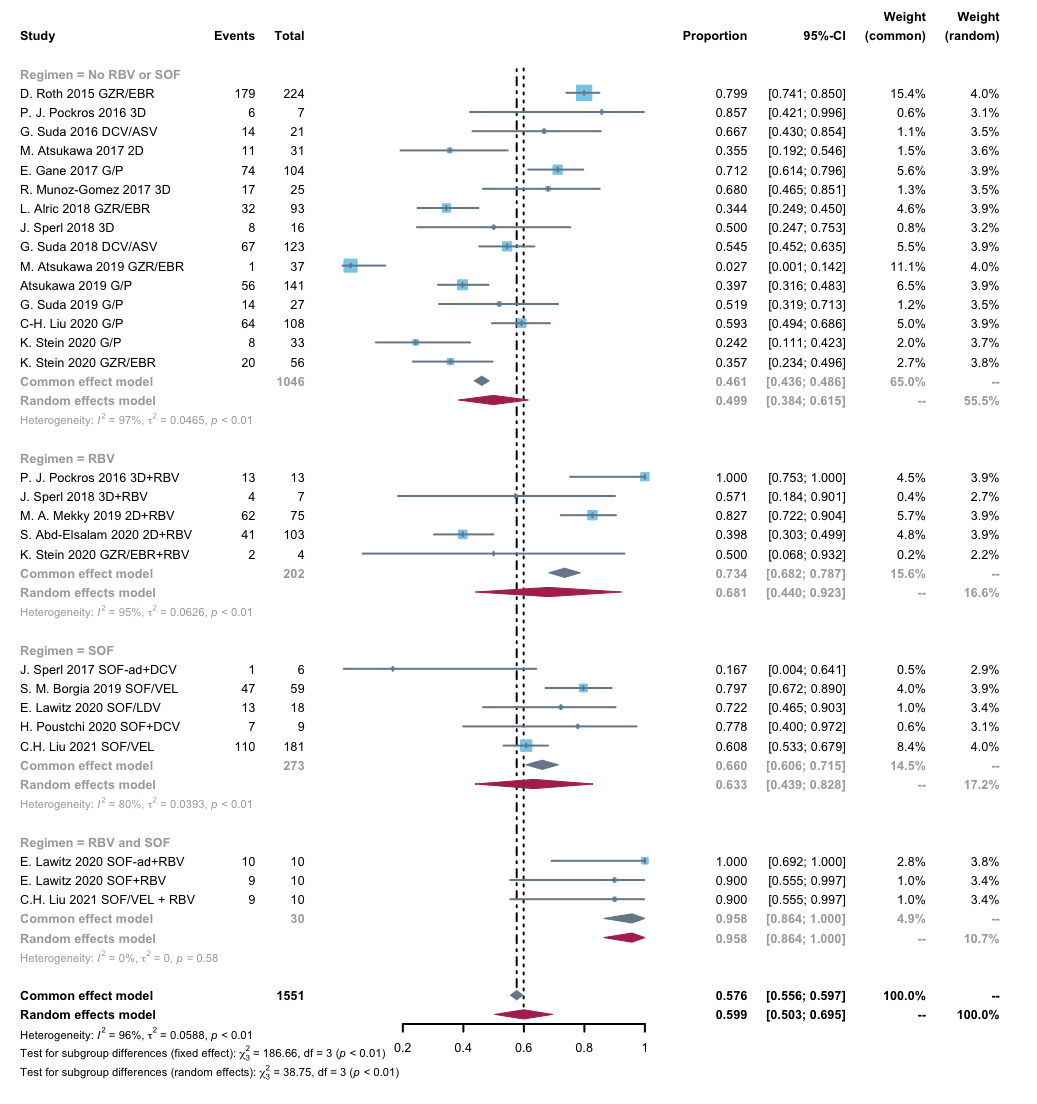
**

**Supplement Figure 7.** The AEs of DAA regimens uncontained RBV or SOF vs regimens with RBV or/and SOF. Estimated advent event rate of DAA regimens uncontained RBV or SOF vs regimens with RBV or/and SOF in end-stage renal disease patients with HCV infection. The mean pooled advent events per regimen with 95% CrI. ASV, asunaprevir; DCV, daclatasvir; DSV, dasabuvir; G/P, glecaprevir /pibrentasvir; GZR/EBR, grazoprevir-elbasvir; LDV, ledipasvir; OBV, ombitasvir; PTV/R, paritaprevir/ritonavir; RBV, ribavirin; SOF, sofosbuvir; VEL, velpatasvir.
