# Supplementary material for: The efficacy and safety of direct-acting antiviral regimens for end-stage renal disease patients with HCV infection: a systematic review and network meta-analysis
Source: Front Public Health. 2023 Sep 29;11:1179531. doi: 10.3389/fpubh.2023.1179531 (PMC10570741; doi:10.3389/fpubh.2023.1179531)
Supplement: Supplementary file 1 [file Data_Sheet_1.zip › Supplementary File 1.docx]

***Supplementary Material***

**The** **Efficacy and** **Safety of Direct-acting Antiviral regimens for end-stage renal disease patients with HCV infection: A Systematic review and Network meta-analysis**

**Ruo Chan Chen1 †, Yinghui Xiong1 †,Yanyang Zeng1, Xiaolei Wang2, Yinzong Xiao3, Yixiang Zheng 1***

*** Correspondence:** Yixiang Zheng, yxzheng@csu.edu.cn

**Supplementary file 1**

**Searching strategy**

We searched 5 databases (PubMed, Ovid (BIOSIS Previews), Embase, Cochrane library and Web of Science) to identify all studies conducted in end-stage renal disease patients with HCV infection. The search strategy included the following terms or synonyms: (chronic) hepatitis C, end-stage renal disease, renal impairment, Chronic kidney disease, clinical trial (Cochrane sensitivity-maximizing search), and pragmatic trial. The year 2004 was set as a limitation because there is no data about DAAs prior to 2004. We performed the first search on April 1^st^, 2021 and updated the search of the databases with modified search strategy on August 18, 2021.

The latest update on August 07, 2023.

- **The first search up to April 1^st^, 2021. Search strategy with items found are shown below:**

**Pubmed (245):**

((((((DAA[Title/Abstract]) OR (DAAs[Title/Abstract])) OR (direct acting antivirals[Title/Abstract])) (((HCV[Title/Abstract]) OR (hepatitis C[Title/Abstract])) OR (hepatitis C[MeSH Major Topic]))) AND (("2004/01/01"[Date - Publication] : "2021/04/01"[Date - Publication]))) AND ((((((end-stage renal disease[Title/Abstract])) OR (renal impairment[Title/Abstract])) OR (chronic kidney disease[Title/Abstract])) OR (renal failure[Title/Abstract])) OR (kidney failure[Title/Abstract]))) AND (((DAA[Title/Abstract]) OR (DAAs[Title/Abstract])) OR (direct acting antivirals[Title/Abstract]))

**Ovid (251) (**All Ovid Journals (Abstracts Only), Search Full Text of Medical University of Central South University of Ovid, BIOSIS Previews 2003, Ovid MEDLINE(R) ALL)**:**

(DAA or DAAs or direct acting antivirals).ab. and (hepatitis C or HCV).ab. and (end-stage renal disease or renal impairment or chronic kidney disease or renal failure or kidney failure).ab.

**Embase (477):**

('end stage renal disease':ab,ti OR 'renal impairment':ab,ti OR 'chronic kidney disease':ab,ti OR 'renal failure':ab,ti OR 'kidney failure':ab,ti). AND（hcv:ab,ti OR 'hepatitis c':ab,ti）AND (daa:ab,ti OR daas:ab,ti OR 'direct acting antivirals':ab,ti)

**Web science (218):**

**(end stage renal disease**(Abstract)**or****renal impairment** (Abstract) **or chronic kidney disease**(Abstract) o**r renal failure** (Abstract) o**r****kidney failure** (Abstract)) AND (**HCV** (Abstract) **or hepatitis C**(Abstract)) AND (**DAA** (Abstract)**or DAAs** (Abstract)**or****direct acting antivirals**(Abstract))

**Cochrane Library (52)**: with Cochrane Library publication date from Jan 2004 to Apr 2021, (Word variations have been searched)

((DAA):ti,ab,kw OR (DAAs):ti,ab,kw OR (direct acting antivirals):ti,ab,kw (Word variations have been searched). ) AND ((HCV):ti,ab,kw OR (hepatitis C):ti,ab,kw (Word variations have been searched)) AND ((end stage renal disease):ti,ab,kw OR (renal impairment):ti,ab,kw OR (chronic kidney disease):ti,ab,kw OR (renal failure):ti,ab,kw OR (kidney failure):ti,ab,kw)

- **Update the search of databases up to August 18, 2021 with ameliorated search strategy. The detail are shown below：**

**Pubmed (N = 531):**

| #1 | Search: ((HCV[Title/Abstract]) OR (hepatitis C[Title/Abstract])) OR (Hepatitis C virus[Title/Abstract]) Sort by: Most Recent | [91,904](https://pubmed.ncbi.nlm.nih.gov/?term=%28%28HCV%5BTitle%2FAbstract%5D%29+OR+%28hepatitis+C%5BTitle%2FAbstract%5D%29%29+OR+%28Hepatitis+C+virus%5BTitle%2FAbstract%5D%29&sort=date) |
| --- | --- | --- |
| #2 | (((((((((((((((DAAs[Title/Abstract]) OR (DAA[Title/Abstract])) OR (direct acting antivirals[Title/Abstract])) OR (sofosbuvir[Title/Abstract])) OR (simeprevir[Title/Abstract])) OR (Grazoprevir[Title/Abstract])) OR (elbasvir[Title/Abstract])) OR (daclatasvir[Title/Abstract])) OR (asunaprevir[Title/Abstract])) OR (ombitasvir[Title/Abstract])) OR (paritaprevir[Title/Abstract])) OR (ritonavir[Title/Abstract])) OR (Glecaprevir[Title/Abstract])) OR (Pibrentasvir[Title/Abstract])) OR (dasabuvir[Title/Abstract])) OR (ledipasvir[Title/Abstract]) | [16,454](https://pubmed.ncbi.nlm.nih.gov/?term=%28%28%28%28%28%28%28%28%28%28%28%28%28%28%28DAAs%5BTitle%2FAbstract%5D%29+OR+%28DAA%5BTitle%2FAbstract%5D%29%29+OR+%28direct+acting+antivirals%5BTitle%2FAbstract%5D%29%29+OR+%28sofosbuvir%5BTitle%2FAbstract%5D%29%29+OR+%28simeprevir%5BTitle%2FAbstract%5D%29%29+OR+%28Grazoprevir%5BTitle%2FAbstract%5D%29%29+OR+%28elbasvir%5BTitle%2FAbstract%5D%29%29+OR+%28daclatasvir%5BTitle%2FAbstract%5D%29%29+OR+%28asunaprevir%5BTitle%2FAbstract%5D%29%29+OR+%28ombitasvir%5BTitle%2FAbstract%5D%29%29+OR+%28paritaprevir%5BTitle%2FAbstract%5D%29%29+OR+%28ritonavir%5BTitle%2FAbstract%5D%29%29+OR+%28Glecaprevir%5BTitle%2FAbstract%5D%29%29+OR+%28Pibrentasvir%5BTitle%2FAbstract%5D%29%29+OR+%28dasabuvir%5BTitle%2FAbstract%5D%29%29+OR+%28ledipasvir%5BTitle%2FAbstract%5D%29&sort=date) |
| #3 | ((((((((((chronic kidney disease[Title/Abstract])) OR (end-stage renal disease[Title/Abstract])) OR (renal impairment[Title/Abstract])) OR (kidney failure[Title/Abstract])) OR (renal failure[Title/Abstract])) OR (renal dysfunction[Title/Abstract])) OR (renal insufficiency[Title/Abstract])) OR (CKD[Title/Abstract])) OR (dialysis[Title/Abstract])) OR (hemodialysis[Title/Abstract]) Sort by: Most Recent | [323,096](https://pubmed.ncbi.nlm.nih.gov/?term=%28%28%28%28%28%28%28%28%28%28chronic+kidney+disease%5BTitle%2FAbstract%5D%29%29+OR+%28end-stage+renal+disease%5BTitle%2FAbstract%5D%29%29+OR+%28renal+impairment%5BTitle%2FAbstract%5D%29%29+OR+%28kidney+failure%5BTitle%2FAbstract%5D%29%29+OR+%28renal+failure%5BTitle%2FAbstract%5D%29%29+OR+%28renal+dysfunction%5BTitle%2FAbstract%5D%29%29+OR+%28renal+insufficiency%5BTitle%2FAbstract%5D%29%29+OR+%28CKD%5BTitle%2FAbstract%5D%29%29+OR+%28dialysis%5BTitle%2FAbstract%5D%29%29+OR+%28hemodialysis%5BTitle%2FAbstract%5D%29&sort=date) |
| #4 | #1AND #2 AND #3 | [531](https://pubmed.ncbi.nlm.nih.gov/?term=%28%28%28%28%28%28%28%28%28%28%28%28chronic+kidney+disease%5BTitle%2FAbstract%5D%29%29+OR+%28end-stage+renal+disease%5BTitle%2FAbstract%5D%29%29+OR+%28renal+impairment%5BTitle%2FAbstract%5D%29%29+OR+%28kidney+failure%5BTitle%2FAbstract%5D%29%29+OR+%28renal+failure%5BTitle%2FAbstract%5D%29%29+OR+%28renal+dysfunction%5BTitle%2FAbstract%5D%29%29+OR+%28renal+insufficiency%5BTitle%2FAbstract%5D%29%29+OR+%28CKD%5BTitle%2FAbstract%5D%29%29+OR+%28dialysis%5BTitle%2FAbstract%5D%29%29+OR+%28hemodialysis%5BTitle%2FAbstract%5D%29%29+AND+%28%28%28%28%28%28%28%28%28%28%28%28%28%28%28%28DAAs%5BTitle%2FAbstract%5D%29+OR+%28DAA%5BTitle%2FAbstract%5D%29%29+OR+%28direct+acting+antivirals%5BTitle%2FAbstract%5D%29%29+OR+%28sofosbuvir%5BTitle%2FAbstract%5D%29%29+OR+%28simeprevir%5BTitle%2FAbstract%5D%29%29+OR+%28Grazoprevir%5BTitle%2FAbstract%5D%29%29+OR+%28elbasvir%5BTitle%2FAbstract%5D%29%29+OR+%28daclatasvir%5BTitle%2FAbstract%5D%29%29+OR+%28asunaprevir%5BTitle%2FAbstract%5D%29%29+OR+%28ombitasvir%5BTitle%2FAbstract%5D%29%29+OR+%28paritaprevir%5BTitle%2FAbstract%5D%29%29+OR+%28ritonavir%5BTitle%2FAbstract%5D%29%29+OR+%28Glecaprevir%5BTitle%2FAbstract%5D%29%29+OR+%28Pibrentasvir%5BTitle%2FAbstract%5D%29%29+OR+%28dasabuvir%5BTitle%2FAbstract%5D%29%29+OR+%28ledipasvir%5BTitle%2FAbstract%5D%29%29%29+AND+%28%28%28HCV%5BTitle%2FAbstract%5D%29+OR+%28hepatitis+C%5BTitle%2FAbstract%5D%29%29+OR+%28Hepatitis+C+virus%5BTitle%2FAbstract%5D%29%29&sort=date) |

**Ovid**（Books@Ovid August 16, 2021,Journals@Ovid Full Text August 17, 2021,  MD&D Ovid Journals,  Derwent Drug File 1964 to 2021 Week 31,  Embase 1974 to 2021 August 16 ,  Ovid MEDLINE(R) ALL 1946 to August 16, 2021）**N=2016**

| Search history sorted by search number ascending | | | |  |
| --- | --- | --- | --- | --- |
|  | [# ▼](https://ovidsp.dc2.ovid.com/ovid-a/ovidweb.cgi?&S=OBJAFPKIJFEBDOOFIPOJGFPEFEALAA00&Sort+Sets=ascending) | **Searches** | **Results** |  |
|  |  |  |  |  |
|  | 33 | 4 and 21 and 32 | 2016 |  |
|  |  |  |  |  |
|  | 32 | 22 or 23 or 24 or 25 or 26 or 27 or 28 or 29 or 30 or 31 | 810036 |  |
|  |  |  |  |  |
|  | 31 | hemodialysis.ab,at. | 157901 |  |
|  |  |  |  |  |
|  | 30 | dialysis.ab,at. | 279482 |  |
|  |  |  |  |  |
|  | 29 | CKD.ab,at. | 103200 |  |
|  |  |  |  |  |
|  | 28 | renal insufficiency.ab,at. | 55305 |  |
|  |  |  |  |  |
|  | 27 | renal dysfunction.ab,at. | 56075 |  |
|  |  |  |  |  |
|  | 26 | renal failure.ab,at. | 224507 |  |
|  |  |  |  |  |
|  | 25 | kidney failure.ab,at. | 17395 |  |
|  |  |  |  |  |
|  | 24 | renal impairment.ab,at. | 39377 |  |
|  |  |  |  |  |
|  | 23 | end-stage renal disease.ab,at. | 90428 |  |
|  |  |  |  |  |
|  | 22 | chronic kidney disease.ab,at. | 149777 |  |
|  |  |  |  |  |
|  | 21 | 5 or 6 or 7 or 8 or 9 or 10 or 11 or 12 or 13 or 14 or 15 or 16 or 17 or 18 or 19 or 20 | 56633 |  |
|  |  |  |  |  |
|  | 20 | ledipasvir.ab,at. | 5107 |  |
|  |  |  |  |  |
|  | 19 | dasabuvir.ab,at. | 2333 |  |
|  |  |  |  |  |
|  | 18 | Pibrentasvir.ab,at. | 1249 |  |
|  |  |  |  |  |
|  | 17 | Glecaprevir.ab,at. | 1266 |  |
|  |  |  |  |  |
|  | 16 | ritonavir.ab,at. | 28214 |  |
|  |  |  |  |  |
|  | 15 | paritaprevir.ab,at. | 2601 |  |
|  |  |  |  |  |
|  | 14 | ombitasvir.ab,at. | 2714 |  |
|  |  |  |  |  |
|  | 13 | asunaprevir.ab,at. | 1542 |  |
|  |  |  |  |  |
|  | 12 | daclatasvir.ab,at. | 4923 |  |
|  |  |  |  |  |
|  | 11 | elbasvir.ab,at. | 1465 |  |
|  |  |  |  |  |
|  | 10 | Grazoprevir.ab,at. | 1494 |  |
|  |  |  |  |  |
|  | 9 | simeprevir.ab,at. | 3400 |  |
|  |  |  |  |  |
|  | 8 | sofosbuvir.ab,at. | 11684 |  |
|  |  |  |  |  |
|  | 7 | direct acting antivirals.ab,at. | 8202 |  |
|  |  |  |  |  |
|  | 6 | DAAs.ab,at. | 9181 |  |
|  |  |  |  |  |
|  | 5 | DAA.ab,at. | 14054 |  |
|  |  |  |  |  |
|  | 4 | 1 or 2 or 3 | 249649 |  |
|  |  |  |  |  |
|  | 3 | Hepatitis C virus.ab,at. | 129604 |  |
|  |  |  |  |  |
|  | 2 | hepatitis C.ab,at. | 196096 |  |
|  |  |  |  |  |
|  | 1 | HCV.ab,at. | 183112 |  |
|  |  |  |  |  |

**Embase (1228)**

| #1 AND #2 AND #3 | 1228 |
| --- | --- |
| #3hemodialysis:ab,ti OR dialysis:ab,ti OR ckd:ab,ti OR 'renal insufficiency':ab,ti OR 'renal dysfunction':ab,ti OR 'renal failure':ab,ti OR 'kidney failure':ab,ti OR 'renal impairment':ab,ti OR 'end-stage renal disease':ab,ti OR 'chronic kidney disease':ab,ti | 477793 |
| #2ledipasvir:ab,ti OR dasabuvir:ab,ti OR pibrentasvir:ab,ti OR glecaprevir:ab,ti OR ritonavir:ab,ti OR paritaprevir:ab,ti OR ombitasvir:ab,ti OR asunaprevir:ab,ti OR daclatasvir:ab,ti OR elbasvir:ab,ti OR grazoprevir:ab,ti OR simeprevir:ab,ti OR sofosbuvir:ab,ti OR 'direct acting antivirals':ab,ti OR daas:ab,ti OR daa:ab,ti | 28060 |
| #1hcv:ab,ti OR 'hepatitis c':ab,ti OR 'hepatitis c virus':ab,ti | 147530 |

**Web of Science (N=612)**

|  | Search |  |
| --- | --- | --- |
|  | ((#3) AND #2) AND #1 | 612 |
| #3 | chronic kidney disease (Abstract) or end-stage renal disease (Abstract) or renal impairment (Abstract) or  kidney failure (Abstract) or renal failure (Abstract) or renal dysfunction (Abstract) or renal insufficiency (Abstract) or CKD (Abstract) or dialysis (Abstract) or hemodialysis (Abstract) | 379587 |
| #2 | DAAs (Abstract) or DAA (Abstract) or direct acting antivirals (Abstract) or sofosbuvir (Abstract) or simeprevir (Abstract) or Grazoprevir (Abstract)  or elbasvir (Abstract) or daclatasvir (Abstract) or asunaprevir (Abstract) or ombitasvir (Abstract)  or paritaprevir (Abstract) or ritonavir (Abstract) or Glecaprevir (Abstract) or Pibrentasvir (Abstract)  or dasabuvir (Abstract) or ledipasvir (Abstract) | 18625 |
| #1 | HCV (Abstract) or hepatitis C (Abstract) or Hepatitis C virus (Abstract) | 123072 |

**Cochrane Library (all date N= 52)**

ID Search Hits

#1 (DAA):ti,ab,kw OR (DAAs):ti,ab,kw OR (direct acting antivirals):ti,ab,kw (Word variations have been searched) N=1008

#2 (HCV):ti,ab,kw OR (hepatitis C):ti,ab,kw OR ("hepatitis C virus"):ti,ab,kw (Word variations have been searched) N=14471

#3 (end stage renal disease):ti,ab,kw OR (renal impairment):ti,ab,kw OR (chronic kidney disease):ti,ab,kw OR (renal failure):ti,ab,kw OR (kidney failure):ti,ab,kw (Word variations have been searched) N=36325

#4 #1 AND #2 AND #3 N=52

- **Update the search of databases up to August 07, 2023 with ameliorated search strategy. The detail are shown below：**

**Pubmed (N = 576):**

| #1 | Search: ((HCV[Title/Abstract]) OR (hepatitis C[Title/Abstract])) OR (Hepatitis C virus[Title/Abstract]) Sort by: Most Recent | 97900 |
| --- | --- | --- |
| #2 | (((((((((((((((DAAs[Title/Abstract]) OR (DAA[Title/Abstract])) OR (direct acting antivirals[Title/Abstract])) OR (sofosbuvir[Title/Abstract])) OR (simeprevir[Title/Abstract])) OR (Grazoprevir[Title/Abstract])) OR (elbasvir[Title/Abstract])) OR (daclatasvir[Title/Abstract])) OR (asunaprevir[Title/Abstract])) OR (ombitasvir[Title/Abstract])) OR (paritaprevir[Title/Abstract])) OR (ritonavir[Title/Abstract])) OR (Glecaprevir[Title/Abstract])) OR (Pibrentasvir[Title/Abstract])) OR (dasabuvir[Title/Abstract])) OR (ledipasvir[Title/Abstract]) | 19410 |
| #3 | ((((((((((chronic kidney disease[Title/Abstract])) OR (end-stage renal disease[Title/Abstract])) OR (renal impairment[Title/Abstract])) OR (kidney failure[Title/Abstract])) OR (renal failure[Title/Abstract])) OR (renal dysfunction[Title/Abstract])) OR (renal insufficiency[Title/Abstract])) OR (CKD[Title/Abstract])) OR (dialysis[Title/Abstract])) OR (hemodialysis[Title/Abstract]) Sort by: Most Recent | 344413 |
| #4 | #1AND #2 AND #3 | 576 |

**Ovid**（Books@Ovid August 16, 2021,Journals@Ovid Full Text August 07, 2023,  MD&D Ovid Journals,  Derwent Drug File 1964 to 2021 Week 31,  Embase 1974 to 2023 August 07 ,  Ovid MEDLINE(R) ALL 1946 to August 07, 2023）**N=2560**

| Search history sorted by search number ascending | | | |  |
| --- | --- | --- | --- | --- |
|  | [# ▼](https://ovidsp.dc2.ovid.com/ovid-a/ovidweb.cgi?&S=OBJAFPKIJFEBDOOFIPOJGFPEFEALAA00&Sort+Sets=ascending) | **Searches** | **Results** |  |
|  |  |  |  |  |
|  | 33 | 4 and 21 and 32 | 2016 |  |
|  |  |  |  |  |
|  | 32 | 22 or 23 or 24 or 25 or 26 or 27 or 28 or 29 or 30 or 31 | 810036 |  |
|  |  |  |  |  |
|  | 31 | hemodialysis.ab,at. | 157901 |  |
|  |  |  |  |  |
|  | 30 | dialysis.ab,at. | 279482 |  |
|  |  |  |  |  |
|  | 29 | CKD.ab,at. | 103200 |  |
|  |  |  |  |  |
|  | 28 | renal insufficiency.ab,at. | 55305 |  |
|  |  |  |  |  |
|  | 27 | renal dysfunction.ab,at. | 56075 |  |
|  |  |  |  |  |
|  | 26 | renal failure.ab,at. | 224507 |  |
|  |  |  |  |  |
|  | 25 | kidney failure.ab,at. | 17395 |  |
|  |  |  |  |  |
|  | 24 | renal impairment.ab,at. | 39377 |  |
|  |  |  |  |  |
|  | 23 | end-stage renal disease.ab,at. | 90428 |  |
|  |  |  |  |  |
|  | 22 | chronic kidney disease.ab,at. | 149777 |  |
|  |  |  |  |  |
|  | 21 | 5 or 6 or 7 or 8 or 9 or 10 or 11 or 12 or 13 or 14 or 15 or 16 or 17 or 18 or 19 or 20 | 56633 |  |
|  |  |  |  |  |
|  | 20 | ledipasvir.ab,at. | 5107 |  |
|  |  |  |  |  |
|  | 19 | dasabuvir.ab,at. | 2333 |  |
|  |  |  |  |  |
|  | 18 | Pibrentasvir.ab,at. | 1249 |  |
|  |  |  |  |  |
|  | 17 | Glecaprevir.ab,at. | 1266 |  |
|  |  |  |  |  |
|  | 16 | ritonavir.ab,at. | 28214 |  |
|  |  |  |  |  |
|  | 15 | paritaprevir.ab,at. | 2601 |  |
|  |  |  |  |  |
|  | 14 | ombitasvir.ab,at. | 2714 |  |
|  |  |  |  |  |
|  | 13 | asunaprevir.ab,at. | 1542 |  |
|  |  |  |  |  |
|  | 12 | daclatasvir.ab,at. | 4923 |  |
|  |  |  |  |  |
|  | 11 | elbasvir.ab,at. | 1465 |  |
|  |  |  |  |  |
|  | 10 | Grazoprevir.ab,at. | 1494 |  |
|  |  |  |  |  |
|  | 9 | simeprevir.ab,at. | 3400 |  |
|  |  |  |  |  |
|  | 8 | sofosbuvir.ab,at. | 11684 |  |
|  |  |  |  |  |
|  | 7 | direct acting antivirals.ab,at. | 8202 |  |
|  |  |  |  |  |
|  | 6 | DAAs.ab,at. | 9181 |  |
|  |  |  |  |  |
|  | 5 | DAA.ab,at. | 14054 |  |
|  |  |  |  |  |
|  | 4 | 1 or 2 or 3 | 249649 |  |
|  |  |  |  |  |
|  | 3 | Hepatitis C virus.ab,at. | 129604 |  |
|  |  |  |  |  |
|  | 2 | hepatitis C.ab,at. | 196096 |  |
|  |  |  |  |  |
|  | 1 | HCV.ab,at. | 183112 |  |
|  |  |  |  |  |

**Embase (1396)**

| #1 AND #2 AND #3 | 1396 |
| --- | --- |
| #3hemodialysis:ab,ti OR dialysis:ab,ti OR ckd:ab,ti OR 'renal insufficiency':ab,ti OR 'renal dysfunction':ab,ti OR 'renal failure':ab,ti OR 'kidney failure':ab,ti OR 'renal impairment':ab,ti OR 'end-stage renal disease':ab,ti OR 'chronic kidney disease':ab,ti | 542555 |
| #2ledipasvir:ab,ti OR dasabuvir:ab,ti OR pibrentasvir:ab,ti OR glecaprevir:ab,ti OR ritonavir:ab,ti OR paritaprevir:ab,ti OR ombitasvir:ab,ti OR asunaprevir:ab,ti OR daclatasvir:ab,ti OR elbasvir:ab,ti OR grazoprevir:ab,ti OR simeprevir:ab,ti OR sofosbuvir:ab,ti OR 'direct acting antivirals':ab,ti OR daas:ab,ti OR daa:ab,ti | 32643 |
| #1hcv:ab,ti OR 'hepatitis c':ab,ti OR 'hepatitis c virus':ab,ti | 158162 |

**Web of Science (N=690)**

|  | Search |  |
| --- | --- | --- |
|  | ((#3) AND #2) AND #1 | 690 |
| #3 | chronic kidney disease (Abstract) or end-stage renal disease (Abstract) or renal impairment (Abstract) or  kidney failure (Abstract) or renal failure (Abstract) or renal dysfunction (Abstract) or renal insufficiency (Abstract) or CKD (Abstract) or dialysis (Abstract) or hemodialysis (Abstract) | 495335 |
| #2 | DAAs (Abstract) or DAA (Abstract) or direct acting antivirals (Abstract) or sofosbuvir (Abstract) or simeprevir (Abstract) or Grazoprevir (Abstract)  or elbasvir (Abstract) or daclatasvir (Abstract) or asunaprevir (Abstract) or ombitasvir (Abstract)  or paritaprevir (Abstract) or ritonavir (Abstract) or Glecaprevir (Abstract) or Pibrentasvir (Abstract)  or dasabuvir (Abstract) or ledipasvir (Abstract) | 22816 |
| #1 | HCV (Abstract) or hepatitis C (Abstract) or Hepatitis C virus (Abstract) | 151243 |

**Cochrane Library (all date N= 56)**

ID Search Hits

#1 (DAA):ti,ab,kw OR (DAAs):ti,ab,kw OR (direct acting antivirals):ti,ab,kw (Word variations have been searched) N=1036

#2 (HCV):ti,ab,kw OR (hepatitis C):ti,ab,kw OR ("hepatitis C virus"):ti,ab,kw (Word variations have been searched) N=15601

#3 (end stage renal disease):ti,ab,kw OR (renal impairment):ti,ab,kw OR (chronic kidney disease):ti,ab,kw OR (renal failure):ti,ab,kw OR (kidney failure):ti,ab,kw (Word variations have been searched) N=42143

#4 #1 AND #2 AND #3 N=56
