# Supplementary material for: The efficacy and safety of direct-acting antiviral regimens for end-stage renal disease patients with HCV infection: a systematic review and network meta-analysis
Source: Front Public Health. 2023 Sep 29;11:1179531. doi: 10.3389/fpubh.2023.1179531 (PMC10570741; doi:10.3389/fpubh.2023.1179531)
Supplement: Supplementary file 1 [file Data_Sheet_1.zip › Supplementary File 2.docx]

***Supplementary Material***

**The** **Efficacy and** **Safety of Direct-acting Antiviral regimens for end-stage renal disease patients with HCV infection: A Systematic review and Network meta-analysis**

**Ruo Chan Chen1 †, Yinghui Xiong1 †,Yanyang Zeng1, Xiaolei Wang2, Yinzong Xiao3, Yixiang Zheng 1***

*** Correspondence:** Yixiang Zheng, yxzheng@csu.edu.cn

# Supplementary file 2

**Quality assessment：**

**Cohort:** NEWCASTLE - OTTAWA QUALITY ASSESSMENT SCALE COHORT STUDIES

| **Study** | | **Selection** | | | | **Comparability** | **Outcome** | | |
| --- | --- | --- | --- | --- | --- | --- | --- | --- | --- |
|  |  | Representati-veness of the exposed cohort | Selection of the non-exposed cohort | Ascertain-ment of exposure | Outcome of interest was not present at start of study | Comparability of cohorts on the basis of the design or analysis | Assessment of outcome | follow-up long enough for outcomes to occur | Adequacy of follow up of cohorts |
| Y. Kawakami | 2016 | * | — | * | * | — | * | * | * |
| R. Miyazaki | 2016 | * | — | * | * | — | * | * | * |
| G. Suda | 2016 | * | — | * | * | — | * | * | * |
| S. Abad | 2017 | * | — | * | * | — | * | * | * |
| S. K. Agarwal, | 2017 | * | * | * | * | — | * | * | * |
| M. Atsukawa | 2017 | * | — | * | * | — | * | * | * |
| N. Morisawa | 2017 | * | — | * | * | — | * | * | * |
| R. Munoz-Gomez | 2017 | * | * | * | * | — | * | * | * |
| T. Otsuka | 2017 | * | — | * | * | — | * | * | * |
| J. Sperl | 2017 | * | — | * | * | — | * | * | * |
| L. Alric | 2018 | * | — | * | * | — | * | * | * |
| A. A. Butt | 2018 | * | * | * | * | — | * | * | * |
| H. Fujii | 2018 | * | — | * | * | — | * | * | * |
| A. Gupta | 2018 | * | * | * | * | — | * | * | * |
| K. Manoj | 2018 | * | * | * | * | — | * | * | * |
| E. Ogawa | 2018 | * | — | * | * | — | * | * | * |
| F. M. Sanai | 2018 | * | * | * | * | — | * | * | * |
| J. Sperl | 2018 | * | * | * | * | — | * | * | * |
| G. Suda | 2018 | * | — | * | * | — | * | * | * |
| S. Taneja | 2018 | * | — | * | * | — | * | * | * |
| M. Atsukawa | 2019 | * | — | * | * | — | * | * | * |
| Atsukawa | 2019 | * | — | * | * | — | * | * | * |
| N. N. Aydin | 2019 | * | — | * | * | — | * | * | * |
| N. Butt | 2019 | * | — | * | * | — | * | * | * |
| A. Y. Elmowafy | 2019 | * | * | * | * | — | * | * | * |
| A. Goel | 2019 | * | — | * | * | — | * | * | * |
| F. Maduell | 2019 | * | * | * | * | — | * | * | * |
| M. A. Mekky | 2019 | * | — | * | * | — | * | * | * |
| G. Suda | 2019 | * | — | * | * | — | * | * | * |
| B. Tatar | 2019 | * | * | * | * | — | * | * | * |
| S. Yaraş | 2019 | * | * | * | * | — | * | * | * |
| S. Abd-Elsalam | 2020 | * | — | * | * | — | * | * | * |
| D. T. Choi | 2020 | * | — | * | * | — | * | * | * |
| P. Debnath | 2020 | * | * | * | * | — | * | * | * |
| R. Eletreby | 2020 | * | * | * | * | — | * | * | * |
| N. Gaur | 2020 | * | — | * | * | — | * | * | * |
| K. Gohel | 2020 | * | * | * | * | — | * | * | * |
| C. Li | 2020 | * | * | * | * | — | * | * | * |
| C.H. Liu | 2020 | * | — | * | * | — | * | * | * |
| C-H. Liu | 2020 | * | — | * | * | — | * | * | * |
| A. Morishita | 2020 | * | — | * | * | — | * | * | * |
| M.Mostafi | 2020 | * | — | * | * | — | * | * |  |
| H. Poustchi | 2020 | * | — | * | * | — | * | * | * |
| H. Y. Seo | 2020 | * | — | * | * | — | * | * | * |
| K. Stein | 2020 | * | * | * | * | — | * | * | * |
| D. Y. H. Yap | 2020 | * | — | * | * | — | * | * | * |
| H. H. Yen | 2020 | * | — | * | * | — | * | * | * |
| M.L.Yu | 2020 | * | * | * | * | — | * | * | * |
| P. N. Cheng | 2021 | * | — | * | * | — | * | * | * |
| C.H. Liu | 2021 | * | * | * | * | — | * | * | * |
| S. Taneja | 2021 | * | — | * | * | — | * | * | * |

*, a study can be awarded a maximum of one star for each numbered item within the Selection and Outcome categories. A maximum of two stars can be given for Comparability.

**Clinical trails：**

| Study | | Clearly stated aim | Inclusion of consecutive patients | Prospective collection of data | Appropriate endpoints | Unbiased assessment of study endpoint | Appropriate follow-up period | Loss to follow up less than 5% | Prospective calculation of study size | Adequate control group | Contem-porary groups | Baseline equivalence of groups | Adequate statistical analyses |
| --- | --- | --- | --- | --- | --- | --- | --- | --- | --- | --- | --- | --- | --- |
| D. Roth | 2015 | ** | ** | ** | ** | ** | ** | ** | ** | ** | ** | ** | ** |
| P. J. Pockros | 2016 | ** | ** | ** | ** | ** | ** | ** | — | — | ** | ** | ** |
| H. Toyoda | 2016 | ** | ** | ** | ** | ** | ** | ** | — | — | ** | ** | ** |
| E. Gane | 2017 | ** | ** | ** | ** | ** | ** | ** | ** | ** | ** | ** | ** |
| H. Kumada | 2018 | ** | ** | ** | ** | ** | ** | ** | — | — | ** | ** | ** |
| S. M. Borgia | 2019 | ** | ** | ** | ** | ** | ** | ** | — | — | ** | ** | ** |
| S. U. R. Cheema | 2019 | ** | ** | ** | ** | ** | ** | ** | ** | ** | ** | ** | ** |
| E. Lawitz-RUBY-I | 2019 | ** | ** | ** | ** | ** | ** | — | — | — | ** | ** | ** |
| E. Lawitz-RUBY-II | 2019 | ** | ** | ** | ** | ** | ** | — | — | — | ** | ** | ** |
| B. S. Lee | 2019 | ** | ** | ** | ** | ** | ** | * | — | — | ** | ** | ** |
| E. Lawitz | 2020 | ** | ** | ** | ** | ** | ** | ** | ** | ** | ** | ** | ** |
| Er. Lawitz | 2020 | ** | ** | ** | ** | ** | ** | ** | ** | ** | ** | ** | ** |

The items are scored 0 (not reported), 1 (reported but inadequate) or 2 (reported and adequate). The global ideal score being 16 for non-comparative studies and 24 for comparative studies
