# Supplementary material for: The efficacy and safety of direct-acting antiviral regimens for end-stage renal disease patients with HCV infection: a systematic review and network meta-analysis
Source: Front Public Health. 2023 Sep 29;11:1179531. doi: 10.3389/fpubh.2023.1179531 (PMC10570741; doi:10.3389/fpubh.2023.1179531)
Supplement: Supplementary file 1 [file Data_Sheet_1.zip › Supplementary Model Code.docx]

***Supplementary Material***

**The** **Efficacy and** **Safety of Direct-acting Antiviral regimens for end-stage renal disease patients with HCV infection: A Systematic review and Network meta-analysis**

**Ruo Chan Chen1 †, Yinghui Xiong1 †,Yanyang Zeng1, Xiaolei Wang2, Yinzong Xiao3, Yixiang Zheng 1***

*** Correspondence:** Yixiang Zheng, yxzheng@csu.edu.cn

# Supplementary Model Code

WinBUGS syntax

This supplementary file shows the Winbugs code, the following components are shown:

The full model the code for networks to connect all-oral DAAs regimens for pooled estimates of SVR ,and rank of the regions:

# Binomial likelihood, logit link

# Joint baseline and treat effects model for multi-arm trials

model{ # *** PROGRAM STARTS

for(i in 1:ns){ # LOOP THROUGH STUDIES

w[i,1] <- 0 # adjustment for multi-arm trials is zero for control arm

delta[i,1] <- 0 # treatment effect is zero for control arm

mu[i] ~ dnorm(m,tau.m) # model for trial baselines re treatment 1

for (k in 1:na[i]) { # LOOP THROUGH ARMS

r[i,k] ~ dbin(p[i,k],n[i,k]) # binomial likelihood

logit(p[i,k]) <- mu[i] + delta[i,k] # model for linear predictor

rhat[i,k] <- p[i,k] * n[i,k] # expected value of the numerators

#Deviance contribution including NAs

dev.NA[i,k] <- 2 * (r[i,k] * (log(r[i,k])-log(rhat[i,k]))

+ (n[i,k]-r[i,k]) * (log(n[i,k]-r[i,k]) - log(n[i,k]-rhat[i,k])))

#Deviance contribution with correction for NAs

dev[i,k] <- dev.NA[i,k]*(1-equals(n[i,1],1))

}

# summed residual deviance contribution for this trial

resdev[i] <- sum(dev[i,1:na[i]])

for (k in 2:na[i]) { # LOOP THROUGH ARMS

# trial-specific LOR distributions

delta[i,k] ~ dnorm(md[i,k],taud[i,k])

# mean of LOR distributions (with multi-arm trial correction)

md[i,k] <- d[t[i,k]] - d[t[i,1]] + sw[i,k]

# precision of LOR distributions (with multi-arm trial correction)

taud[i,k] <- tau *2*(k-1)/k

# adjustment for multi-arm RCTs

w[i,k] <- (delta[i,k] - d[t[i,k]] + d[t[i,1]])

# cumulative adjustment for multi-arm trials

sw[i,k] <- sum(w[i,1:k-1])/(k-1)

}

}

totresdev <- sum(resdev[]) # Total Residual Deviance

d[1]<-0 # treatment effect is zero for reference treatment

# vague priors for treatment effects

for (k in 2:nt){ d[k] ~ dnorm(0,.0001) }

sd ~ dunif(0,0.5) # vague prior for between-trial SD

tau <- pow(sd,-2) # between-trial precision = (1/between-trial variance)

mu.new ~ dnorm(m,tau.m) # predictive dist. for baseline (log-odds)

m ~ dnorm(0,.0001) # vague prior for mean (baseline model)

var.m <- 1/tau.m # between-trial variance (baseline model)

tau.m <- pow(sd.m,-2) # between-trial precision = (1/between-trial variance)

sd.m ~ dunif(0,5) # vague prior for between-trial SD (baseline model)

#sd.m <- sqrt(var.m)

#tau.m ~ dgamma(0.001,0.001)

# Provide estimates of treatment effects T[k] on the natural (probability) scale

# based on posterior distr of baseline model and T.new[k] based on

# predictive distr of baseline model

for (k in 1:nt) {

logit(T[k]) <- m + d[k]

logit(T.new[k]) <- mu.new + d[k]

}

# pairwise ORs and LORs for all possible pair-wise comparisons

for (c in 1:(nt-1)) {

for (k in (c+1):nt) {

or[c,k] <- exp(d[k] - d[c])

lor[c,k] <- (d[k]-d[c])

}

}

}

R. Code for the the single-rate meta-analysis with random-effects model was used for the pooled estimate and subgroup analysis:

library(meta)

DAA<-safety_data

head(DAA)

ade<-DAA$`Any adverse events`

rate <- transform(DAA, p.p = ade/TN, log = log(ade/TN),

logit = log((ade/TN)/(1 - ade/TN)),

arcsin.size = asin(sqrt(ade/(TN + 1))),

darcsin = 0.5 * (asin(sqrt(ade/(TN + 1)) )) +

asin((sqrt(ade + 1)/(TN + 1))))

shapiro.test(rate$p)

shapiro.test(rate$log)

shapiro.test(rate$logit)

shapiro.test(rate$arcsin.size)

shapiro.test(rate$darcsin)

meta1 <- metaprop(ade, TN, data = DAA,studlab = paste(Author),sm="PRAW",incr=0.5,allincr=TRUE,addincr=FALSE,random = T,subgroup = Intervention)

forest(meta1,digits=3,family="sans",fontsize=9.5,lwd=2,col.diamond.fixed="lightslategray",col.diamond.lines.fixed="lightslategray",

col.diamond.random="maroon",col.diamond.lines.random="maroon",col.square="skyblue",col.study="lightslategray",

lty.fixed=4,plotwidth="8cm",colgap.forest.left="1cm",colgap.forest.right="1cm",just.forest="right",colgap.left="0.5cm", colgap.right="0.5cm")
